# Ultrasound Contrast Agent Needle Priming: Impact on Sonographic Biopsy Needle Visibility in a Porcine Liver Model

**DOI:** 10.1007/s00270-024-03758-1

**Published:** 2024-06-19

**Authors:** Per Thunswärd, Ellen Bergkvist, Liya Vishnevskaya, Yngve Forslin, Håkan Ahlström

**Affiliations:** 1 Department of Radiology, Västmanlands Hospital Västerås, Västerås, Sweden; 2 Radiology, Department of Surgical Sciences, Uppsala University, Uppsala University Hospital, Entrance 70, 1st floor, S-751 85 Uppsala, Sweden; 3 Department of Radiology, Karolinska University Hospital, Stockholm, Sweden; 4 Department of Clinical Science, Intervention and Technology – CLINTEC, Karolinska Institutet, Stockholm, Sweden; 5 Antaros Medical AB, Mölndal, Sweden

**Keywords:** Needle visibility, Ultrasound, Contrast-enhanced ultrasound, Contrast-specific imaging mode, Ultrasound contrast agent, Needle priming, Needle filling, Core needle biopsy, Core biopsy needle

## Abstract

**Purpose:**

The visibility of biopsy needles in contrast-specific imaging mode can be improved by priming them with an ultrasound contrast agent (previously demonstrated in a phantom model/ex vivo). The purpose of this study was to validate this priming method in a porcine in vivo model.

**Materials and Methods:**

Using a small syringe, full-core biopsy needles were primed with sulfur hexafluoride, an ultrasound contrast agent, with non-primed needles serving as controls (*n* = 30 + 30). Liver punctures were performed in a porcine model following intravenous administration of the same ultrasound contrast agent. Needle visibility, both in their entirety and at the tips, was evaluated in split-screen mode using contrast-specific imaging and B-mode (low mechanical index). The assessment included quantitative analysis, calculating the contrast-to-noise ratio, and qualitative evaluation through structured grading by three radiologists.

**Results:**

After needle priming, the contrast-to-noise ratio was superior for the needle in its entirety in contrast-specific imaging mode (*p* < 0.001) and slightly inferior in B-mode (*p* = 0.008). No differences were observed for the needle tips in either imaging mode. Qualitatively, the needle visibility was deemed clinically superior after needle priming throughout in contrast-specific imaging mode (*p* < 0.001), whereas no clinically relevant differences in B-mode for either the needle in its entirety (*p* = 0.11) or the needle tip (*p* = 1) were observed.

**Conclusion:**

In this in vivo porcine liver model experiment, priming biopsy needles with ultrasound contrast agent improved needle visibility in contrast-specific imaging mode but slightly reduced it in B-mode. These findings support the method’s use for biopsies requiring target visualization in contrast-specific imaging mode.

No level of evidence.

**Graphical Abstract:**

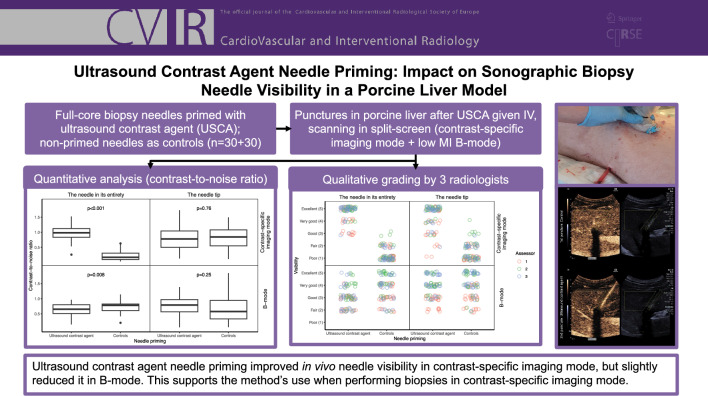

**Supplementary Information:**

The online version contains supplementary material available at 10.1007/s00270-024-03758-1.

## Introduction

Performing biopsies on hepatic lesions with low visibility in B-mode ultrasound (US) or when there is a need to target viable parts within lesions with necrotic components can be advantageously carried out under guidance of contrast-enhanced ultrasound (CEUS) [1–4]. To improve the success rate and reduce the risks of complications, it is important to visualize the trajectory of the needle and the needle tip [5, 6]. In contrast-specific imaging mode (CEUS mode), the needle is sometimes difficult to visualize due to the vivid enhancement of the parenchymatous organs [7, 8]. A common approach is using a split-screen mode, showing one CEUS mode image alongside one B-mode image [1, 2, 7, 8]. When using CEUS mode, the machine is set to a lower mechanical index (MI) to minimize the disruption to the microbubbles in the US contrast agent. As a result, the quality of the low MI B-mode image is inferior compared to conventional B-mode [2]. While needle visibility is often sufficient in split-screen mode [2], challenging clinical situations may still require improved needle visualization [9].

In previous ex vivo and phantom model studies in CEUS mode, the visibility of biopsy needles, coaxial introducer needles, and fine needles improved by priming the needles with an ultrasound contrast agent (USCA) [10, 11]. Improved visibility of a biopsy needle by USCA priming in a single patient has also been described in a letter to the editor [9]. However, there is a need for a systematic in vivo validation before introducing this technique into clinical practice.

## Materials and Methods

Ultrasound-guided punctures were performed in a pig liver, from which recordings were subsequently evaluated quantitatively and qualitatively.

### Porcine Model and Sonographic Prerequisites

**Fig. 1:**
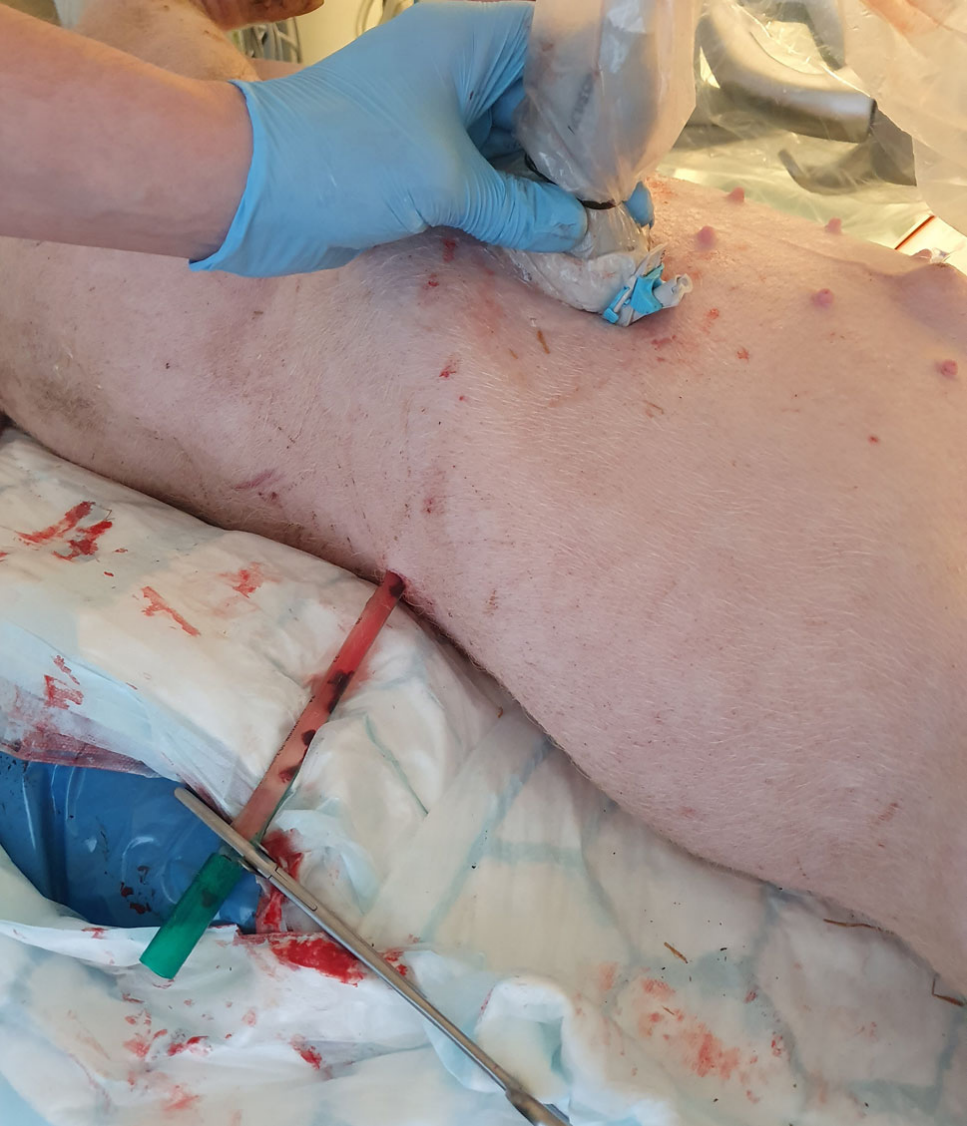
The porcine model. A 10.7 mm/32 F thoracic catheter was inserted into the liver under ultrasound guidance. The catheter (seen in the lower part of the picture) was subsequently filled with saline and clamped

A single male Swedish pig weighing 34 kg was included in this study. The pig was first put under deep anesthesia and prepared (and, ultimately, euthanized) according to the protocol in Online Resource 1. Thereafter, an incision was made in the skin of the right flank, and a thoracic catheter (diameter 10.7 mm/32 Fr; length 40 cm) with an included metal trocar (Vygon, Ecouen, France) was inserted intercostally into the liver under ultrasound guidance to simulate an anechoic target lesion (see Fig. 1). The center point of the catheter had an average depth (min–max) of 4.8 cm (4.1–5.6 cm). The catheter was filled with saline and clamped to prevent air artifacts.

A Siemens ACUSON Sequoia unit (Siemens Medical Solutions, Inc., Mountain View, CA, USA), equipped with a 5C1 curved transducer, was used for US scanning. A guide for 18G needles (Verza, CIVCO Medical Instruments Co., Inc., IA, USA) set at an angle of three (61°) was used, together with its corresponding guiding lines. Imaging and recording were performed in split-screen imaging mode with a CEUS mode image on the left (low-frequency setting) and a low MI B-mode image on the right (mid-frequency setting), at a rate of nine frames per second. The maximum depth was set to 9 cm.

### Biopsy Needle Priming and Puncture Procedure

**Fig. 2:**
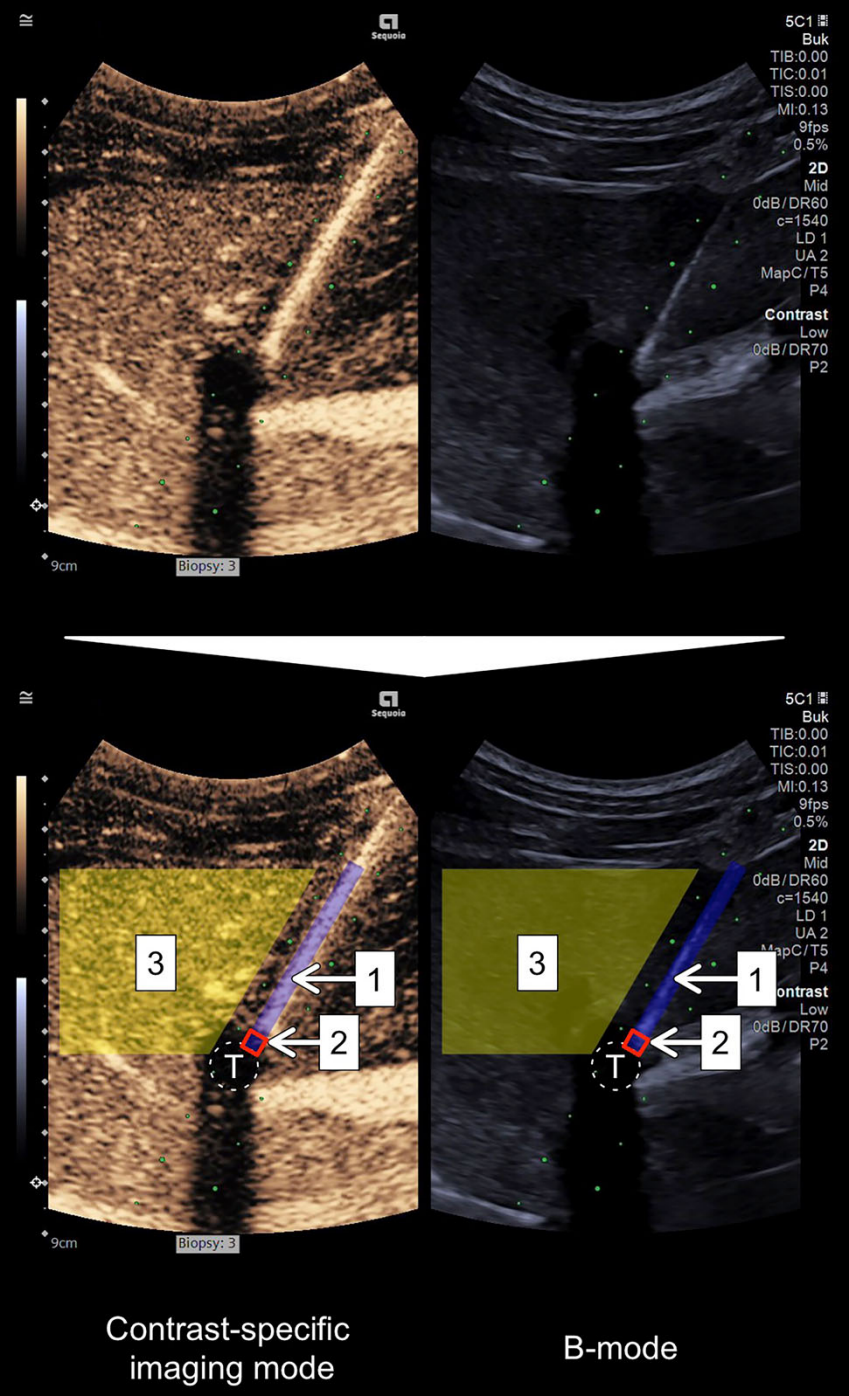
Example of an observation. Top row: image in split-screen mode of a biopsy needle primed with ultrasound contrast agent in its end position reaching the catheter. Bottom row: same image with 1 (blue rectangle with diameter of 3.5 mm): ROI delineating the needle in its entirety; 2 (red square with a side of 3.5 mm): the needle tip; 3 (yellow polygon): area outside ROIs (background) and T (white dashed circle): catheter simulating target lesion

BioPince Ultra Full Core biopsy instruments 18G × 20 cm (Argon Medical Devices, Inc., TX, USA) were primed with 0.6 mL of the USCA sulfur hexafluoride (SonoVue; Bracco SpA, Milan, Italy) using a 1 mL syringe, as described and depicted in detail in our previous paper [10]. After priming and without delay, each biopsy needle was inserted into the liver while aiming at the perpendicularly located thoracic catheter, as illustrated in Fig. 2. The needles were considered to be in the desired end position once the tip reached the target, and a slight movement of the catheter was detected. This was to ensure that a proper delineation of the needle tip could be made when processing the image, regardless of its visibility. Each recording captured the needle insertion and approximately 5 s with the needle in the end position. After complete needle extraction, the biopsy instruments were fired off outside the pig and primed again before the next puncture.

Under ventilator-induced apnea, a total of 60 punctures were performed in ten sets non-blinded by the same operator. Each set included three pairs of punctures, with each pair consisting of a primed needle and a non-primed control. Before initiating each pair of punctures, 1.2 mL of the USCA sulfur hexafluoride was administrated via a peripheral venous catheter in an auricular vein, followed by flushing with 10 mL of 0.9% saline. The USCA dosage was chosen based on the pig’s weight and aligned with the standard dosage given to pediatric patients in our clinic. The first puncture in each pair was performed as soon as the homogeneous contrast uptake could be seen in the liver and the second as soon as possible thereafter. In every other set, the punctures were performed with either the primed needle or the control first, to prevent bias-related to the background intensity decreasing over time. A new entry point and a new primed instrument were used for each new set of punctures.

### Quantitative Evaluation

**Fig. 3:**
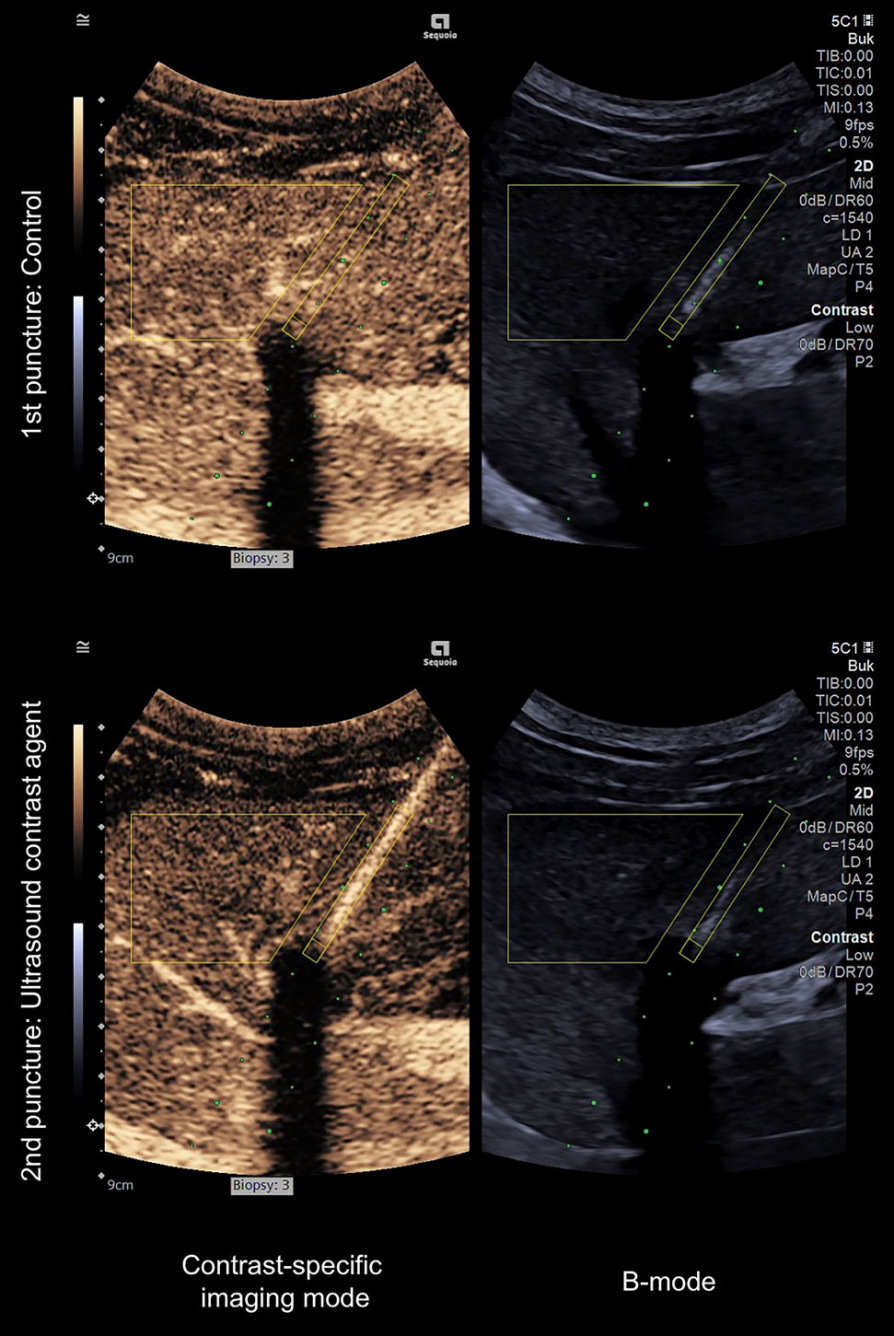
Example of a pair of punctures (third pair of the fifth set) with needles in their end positions adjacent to the thoracic drain. Yellow boxes represent the two regions of interest and the background, as described in detail in Fig. 2. The pair was chosen to represent a contrast-to-noise ratio near the median in contrast-specific imaging mode for the needle in its entirety of the ultrasound contrast agent primed needles

To quantitatively evaluate the needle visibility, the contrast-to-noise ratio (CNR), originally proposed by Patterson and Foster as the contrast-to-speckle ratio, was calculated for each needle in its entirety and each needle tip in both CEUS mode and B-mode, respectively, using:

$${\text{CNR}} = \frac{{\left| {\mu_{{\text{i}}} - \mu_{{\text{o}}} } \right|}}{{\sqrt {\sigma_{{\text{i}}}^{{2}} + \sigma_{{\text{o}}}^{{2}} } }}$$

with *μ*_i_ representing the average signal intensity (SI) inside the region of interest (ROI), *μ*_o_: average SI outside the ROI, *σ*_i_^2^ the SI variance inside the ROI, and *σ*_o_^2^ the SI variance outside the ROI [12, 13]. ROIs were defined for the needles in their entirety (a rectangle) and the needle tips (a square). This was achieved through a combined assessment of the CEUS mode and B-mode images, supported by observing the movements of the needles during insertion, along with those of the thoracic drain upon needle tip contact. The area outside the ROIs was delineated by a polygon representing a segment of the liver parenchyma at the same depth as the intrahepatic course of the biopsy needle. The analysis focused on the image frames capturing each needle tip in its end position touching the thoracic drain (that is, not the insertion phase of the recordings). Examples can be found in Figs. 2 and 3, and all observations are presented in Online Resource 2. The ROI delineation process was carried out in a non-blinded manner.

### Qualitative Evaluation

The recordings (including both the needle insertion and approximately 5 s in the end position) were evaluated in a blinded manner by three radiologists with 9, 15, and 26 years of experience, respectively, including multiyear familiarity with ultrasound-guided punctures. Matching pairs of videos (one primed and one control) were randomly presented side by side via a web interface (all videos are available in Online Resource 3). For both the needles in their entirety and the needle tips, the visibility in CEUS mode and B-mode was evaluated on a Likert scale (1: poor; 2: fair; 3: good; 4: very good; and 5: excellent). The radiologists answered the following two questions in their assessment:

1. The needle in its entirety:
  In which of the two videos is the needle in its entirety most visible?
  Do you consider the difference in visibility to be clinically relevant?
2. The needle tip:
  In which of the two videos is the tip of the needle most visible?
  Do you consider the difference in visibility to be clinically relevant?

### Image Processing and Statistics

The recordings were processed using the application ImageJ version 1.54 g (Wayne Rasband, National Institute of Health, Bethesda, MD, USA). For statistical analyses and rendering of charts, R version 4.3.2 (R Foundation for Statistical Computing, Vienna, Austria) was used. All statistical tests were two-tailed and performed at a significance level of 0.05.

In the quantitative evaluation, the mean CNRs for each puncture were calculated, and medians were derived. A Wilcoxon signed-rank test was used to test the differences in CNRs between the primed and non-primed needles.

For the qualitative evaluation, the results of the Likert scale gradings were illustrated descriptively as jitter plots. Contingency tables (2 × 2) were created to combine the frequencies for the four possible combinations of questions 1 and 2. The primary outcome variable was formed by aggregating the assessors’ weighted assessments by the majority. Comparisons of the frequency of (1) primed biopsy needles with superior visibility independently of clinical relevance and (2) primed biopsy needles with clinically relevant superior visibility were performed using an exact binominal test (hypothesized probability of success of 0.5).

The variations in CNRs and Likert scale visibility for the needles in their entirety across the first, second, and third orders of priming were presented as jitter plots and analyzed with a Friedman test. If the outcome was statistically significant, it was supplemented with a Wilcoxon signed-rank test to compare the priming orders. It was deemed unlikely that needle tip visibility would be affected by repeated priming, which is why evaluation of these was waived.

## Results

### Quantitative Evaluation

**Fig. 4:**
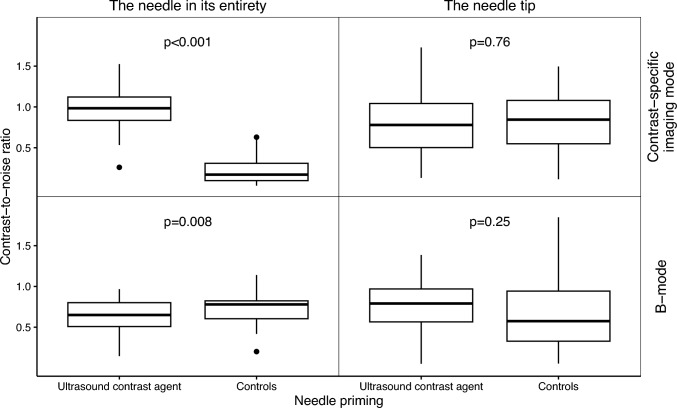
Contrast-to-noise ratio for ultrasound contrast agent primed needles versus controls for both the entire needles and the needle tips contrast-specific imaging mode and B-mode illustrated as box and whisker plot (*n* = 30 + 30). The *p*-values were calculated with a Wilcoxon signed-rank test

After needle priming, the CNR of the needle in its entirety was superior in CEUS mode and, conversely, slightly inferior in B-mode. However, for the needle tip, there were no differences in any of the imaging modes. Detailed results are presented in Fig. 4 and Table 1. Additionally, there were no differences in CNR for the primed needles in their entirety based on the order of priming (see Fig. 6).

**Table 1:** Results of the quantitative evaluation

Imaging mode	ROI	Order of puncture	Median CNR		
			USCA	Controls	*p*^a^
Contrast-specific imaging mode	The needle in its entirety	1	0.86	0.17	*p* = 0.002
		2	0.95	0.13	*p* = 0.002
		3	1.05	0.26	*p* = 0.004
		Aggregated^b^	0.98	0.17	*p* < 0.001
	The needle tip	Aggregated^b^	0.78	0.84	*p* = 0.76
B-mode	The needle in its entirety	1	0.67	0.70	*p* = 0.56
		2	0.57	0.81	*p* = 0.01
		3	0.71	0.78	*p* = 0.13
		Aggregated^b^	0.65	0.78	*p* = 0.008
	The needle tip	Aggregated^b^	0.79	0.58	*p* = 0.25

*CNR* Contrast-to-noise ratio, *ROI* region of interest, *USCA* ultrasound contrast agent

^a^Wilcoxon signed-rank test comparing the USCA-primed needles with the controls

^b^Aggregation of the different order of primings (1–3)

### Qualitative Evaluation

**Fig. 5:**
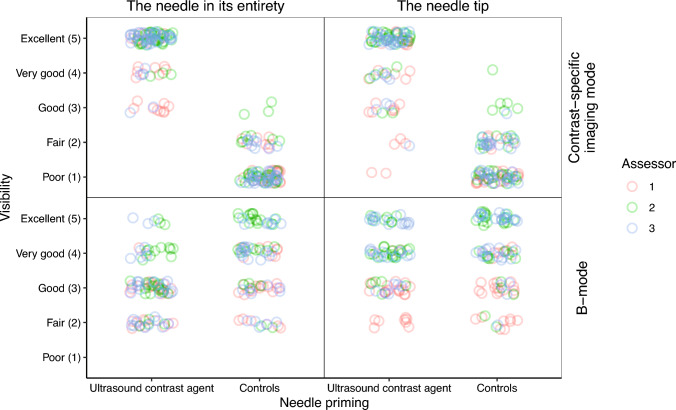
Jitter plot with the distribution of the qualitative grading on the Likert scale for the three different assessors (*n* = 30 + 30)

In CEUS mode, the visibility of both the needle in its entirety and the needle tip was significantly improved after priming, regardless of whether clinical relevance was considered or not (*p*_1_ < 0.001 and *p*_2_ < 0.001). However, in B-mode after priming the needle, the visibility of the needle in its entirety was rated lower when clinical relevance was not considered (*p*_1_ = 0.005), with no statistical difference observed when clinical relevance was taken into account (*p*_2_ = 0.11). There were no statistical differences in the B-mode needle tip visibility after priming, regardless of clinical relevance (*p*_1_ = 0.10 and *p*_2_ = 1). Detailed results are presented in Fig. 5 and Table 2.

**Table 2:** Results of the qualitative evaluation

Contrast-specific imaging mode visibility										
The needle in its entirety	Assessor		1		2		3		Weighted together^a^	
	Priming		USCA	Ctrls	USCA	Ctrls	USCA	Ctrls	USCA	Ctrls
	Clinically	Yes	29	0	30	0	30	0	30	0
	relevant	No	1	0	0	0	0	0	0	0
	*p*_1_		< 0.001		< 0.001		< 0.001		< 0.001	
	*p*_2_		< 0.001		< 0.001		< 0.001		< 0.001	
The needle tip	Assessor		1		2		3		Weighted together^a^	
	Priming		USCA	Ctrls	USCA	Ctrls	USCA	Ctrls	USCA	Ctrls
	Clinically	Yes	26	0	30	0	28	0	28	0
	relevant	No	4	0	0	0	2	0	2	0
	*p*_1_		< 0.001		< 0.001		< 0.001		< 0.001	
	*p*_2_		< 0.001		< 0.001		< 0.001		< 0.001	

B-mode visibility										
The needle in its entirety	Assessor		1		2		3		Weighted together^a^	
	Priming		USCA	Ctrls	USCA	Ctrls	USCA	Ctrls	USCA	Ctrls
	Clinically	Yes	3	9	4	11	2	3	2	8
	relevant	No	4	14	3	12	6	19	5	15
	*p*_1_		0.005		0.005		0.02		0.005	
	*p*_2_		0.15		0.12		1		0.11	
The needle tip	Assessor		1		2		3		Weighted together^a^	
	Priming		USCA	Ctrls	USCA	Ctrls	USCA	Ctrls	USCA	Ctrls
	Clinically	Yes	4	4	4	6	1	2	2	3
	relevant	No	7	15	4	16	12	15	8	17
	*p*_1_		0.20		0.02		0.58		0.10	
	*p*_2_		1		0.75		1		1	

*USCA* Ultrasound contrast agent, *Ctrls* controls, *p*_1_ exact binominal test independently of clinical relevance, *p*_2_ exact binominal test for the subset of clinically relevant observations

^a^By majority

**Fig. 6:**
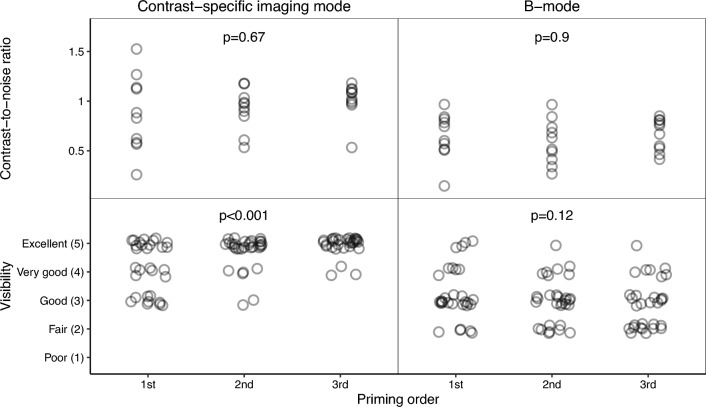
Illustration of the visibility of the needle in its entirety depending on the priming order (*n* = 30). Top row: scatterplot of contrast-to-noise ratio. Bottom row: jitter plot with qualitative grading on the Likert scale. The *p*-values were calculated with a Friedman test

In CEUS mode, there was a difference in the Likert-graded visibility of the primed needles in their entirety based on the priming order (*p* < 0.001), with the second and third orders rated superior to the first (*p* = 0.003 and *p* < 0.001, respectively), but with no difference was noted between the second and third orders (*p* = 0.15). In B-mode, there were no differences in the Likert-graded visibility for the primed needles between the priming orders (*p* = 0.12). The distribution is illustrated in Fig. 6.

## Discussion

In this porcine study, we validated our previously described method for priming full-core biopsy needles. The method, except for the quantitative analysis of the needle tip, was associated with increased CEUS mode needle visibility, and the difference was deemed clinically relevant. Conversely, priming was associated with somewhat impaired B-mode visibility for the needles in their entirety, though not judged clinically relevant. Furthermore, no differences were observed for the needle tips in B-mode, neither quantitatively nor qualitatively. The results regarding the effect on the visibility of the needles in their entirety, depending on their order of priming (first, second, and third), varied, with no statistical difference in the quantitative evaluation for any of the imaging modes, whereas in the qualitative evaluation, the first order was somewhat inferior compared with the second and third orders in CEUS mode.

In CEUS mode, the needles primed with undiluted USCA were distinguishable from the liver parenchyma perfused with a lower concentration of USCA (1.2 mL dispersed in the blood volume). These findings, along with the lack of a marked difference in B-mode needle visibility, align with our previous phantom and ex vivo studies [10, 11]. The marginal reduction in B-mode needle visibility after needle priming may arise from the significantly smaller increase in B-mode echogenicity compared to CEUS mode echogenicity, along with a slight rise in liver echogenicity following intravenous USCA administration. We suggest that the absence of disparities in CNR for the needle tips in both imaging modes might have been partially due to anechoic artifacts adjacent to (and produced by) the thoracic drain, which consequently diminished the signal intensity in the needle tip ROI. The artifact issue could have been prevented by conducting the experiment without an anechogenic target. However, refraining from using a highly echogenic target would reduce the possibility of confirming that the needle tip had reached the intended target and depth, as indicated by the small movement in the drain induced by the needle. Furthermore, a different target that produces fewer artifacts (e.g., a thin-walled polyolefin hose, as used in our previous phantom study) could have been utilized [10]. However, we did not consider it practically possible in vivo. The variations in the needle tip outcome between the qualitative and quantitative analyses might have stemmed from the quantitative analysis solely incorporating image frames at the end position, whereas the qualitative analysis considered the entire trajectory of the needle from the skin surface to the end position, thus not being as affected by the thoracic drain artifacts. Overall, we estimate that no significant influence on needle visualizability in CEUS mode should have been overlooked, given the otherwise consistent results in CEUS mode. Unlike our previous experiments on slaughtered bovine liver, no puncture-related artifacts were observed, despite six punctures at the same site in each of the ten sets.

Our study was limited to a specific kind of biopsy needle, one ultrasound machine, and one transducer with a fixed puncture angle. The use of a porcine model is advantageous based on its great similarities with humans, albeit not perfect. The limitation arises from the significantly lower body weight of the included pig compared to that of an adult human, together with the small percentage of body fat, which was not particularly challenging (as often is the case in the clinical setting). Furthermore, the average depth of the simulated target (barely 5 cm) may be considered rather superficial compared to many clinical cases. The B-mode imaging of the split-screen mode image is often used in the clinical setting when performing biopsies in CEUS mode, and therefore the most relevant to evaluate. However, at the same time, the needle visibility results become less generalizable for “ordinary” B-mode, given the lower MI used in split-screen mode. Finally, conducting both the punctures and ROI delineation in a non-blinded manner introduces a potential bias, which is a weakness.

## Conclusion

In this in vivo experiment conducted on a porcine liver, our technique of priming biopsy needles with USCA improved needle visibility in CEUS mode, albeit slightly reducing it in B-mode. These findings support the use of the priming method during biopsy procedures that require target visualization in CEUS mode.

### Supplementary Information

Below is the link to the electronic supplementary material.

Supplementary file1 (PDF 65 KB)

Supplementary file2 (PDF 9025 KB)

Supplementary file3 (PDF 176759 KB)
