# Supplementary material for: Ultrasound Contrast Agent Needle Priming: Impact on Sonographic Biopsy Needle Visibility in a Porcine Liver Model
Source: Cardiovasc Intervent Radiol. 2024 Jun 19;47(7):1000–8. doi: 10.1007/s00270-024-03758-1 (PMC11239778; doi:10.1007/s00270-024-03758-1)
Supplement: Supplementary file 1 — Supplementary file1 (PDF 65 KB) [file 270_2024_3758_MOESM1_ESM.pdf]

Online Resource 1: Protocol for anesthesia, preparation and termination of the experimental animal

Anesthesia and preparations:

- Induction into general anesthesia by injection of xylazine (Rompun®, Bayer A/S, Denmark) and tiletamin/zolzepam (Zoletil 100®, Virbac S.A., France) subcutaneously
- Placement of the animal in a supine position
- Establishment of venous access
- Maintenance of anesthesia by a single dose of 20 mg morphine (Pfizer, Sollentuna, Sweden) and 100 mg ketamine hydrochloride (Pfizer), followed by intravenous infusion of ketamine at a rate of 30 mg/kg/h (Ketaminol vet, Intervet AB, Sweden), along with fentanyl (Fentanyl, B Braun Medical AB, Sweden) at a rate of 0.04 mg/kg/h, and midazolam (Midazolam Hameln, AlgolPharma AB, Sweden) at a rate of 0.1 mg/kg/h
- Obtaining muscle blockade through intravenous administration of rocuronium (Esmeron, MSD AB, Sweden) at a rate of 2.5 mg/kg/h
- Ventilation through a surgically induced tracheostomy
- Insertion of a central venous catheter into the right internal jugular vein
- Catheterization of the urinary bladder

Termination:

- After the experiment, euthanasia was performed with intravenous administration of 100 mmol KCl
- Death was confirmed by verifying the cessation of cardiac activity (straight ECG curve) and the absence of variations on the blood pressure curve
