# Supplementary material for: Ultrasound Contrast Agent Needle Priming: Impact on Sonographic Biopsy Needle Visibility in a Porcine Liver Model
Source: Cardiovasc Intervent Radiol. 2024 Jun 19;47(7):1000–8. doi: 10.1007/s00270-024-03758-1 (PMC11239778; doi:10.1007/s00270-024-03758-1)
Supplement: Supplementary file 2 — Supplementary file2 (PDF 9025 KB) [file 270_2024_3758_MOESM2_ESM.pdf]

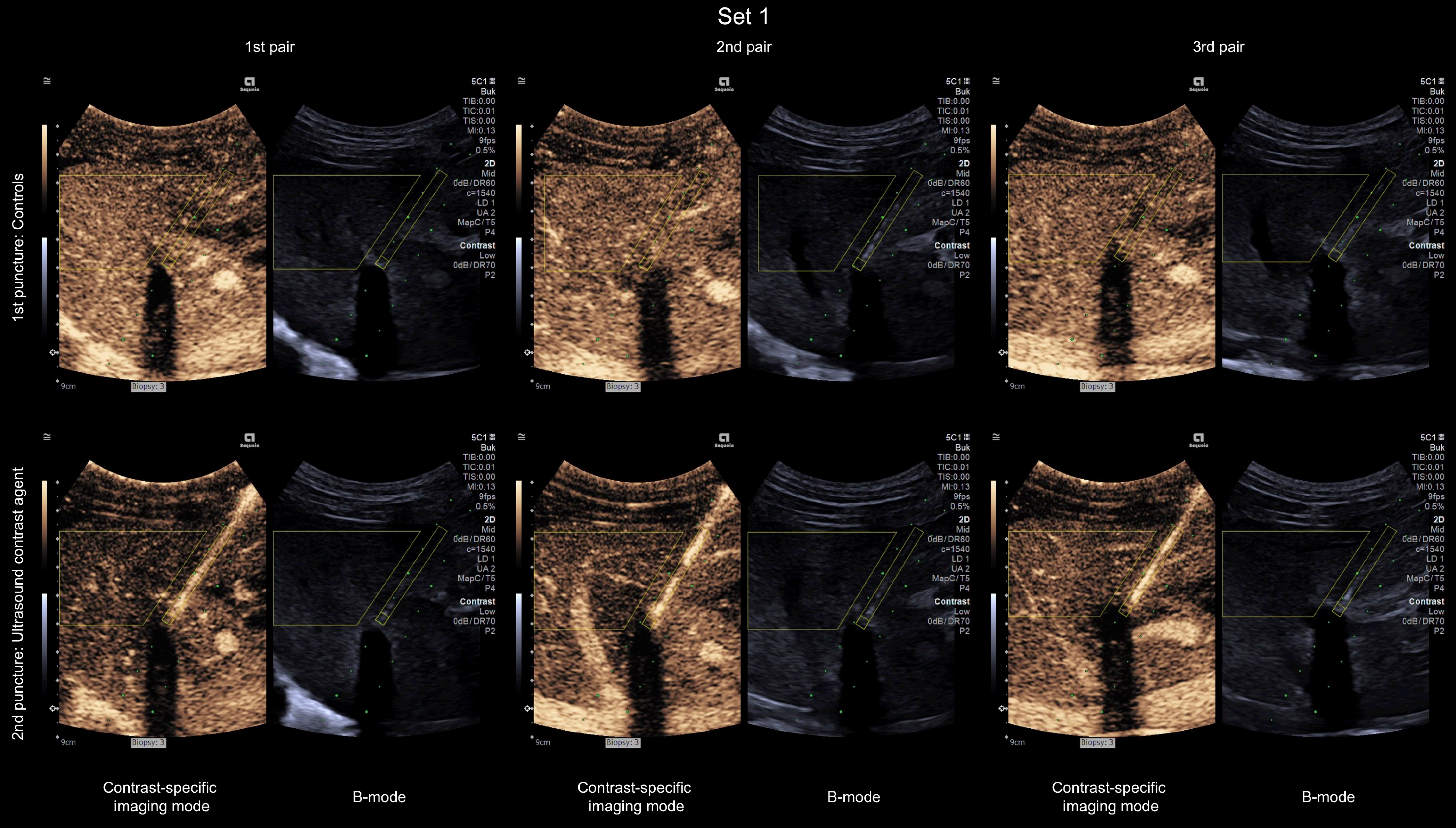
Set 1
1st pair
2nd pair
3rd pair
1st puncture: Controls
2nd puncture: Ultrasound contrast agent
Contrast-specific imaging mode
B-mode
Contrast-specific imaging mode
B-mode
Contrast-specific imaging mode
B-mode

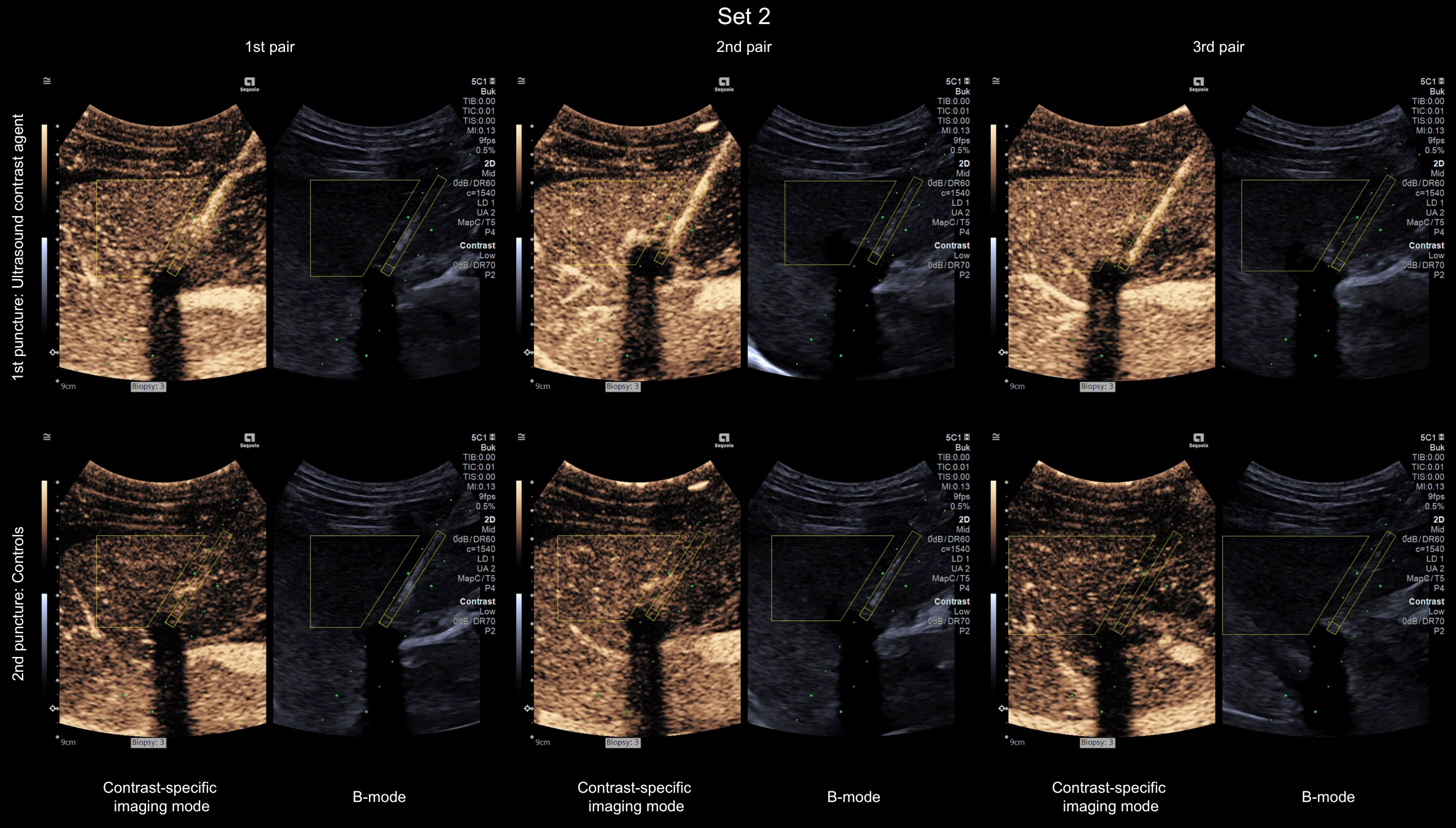
Set 2
1st pair
2nd pair
3rd pair
1st puncture: Ultrasound contrast agent
2nd puncture: Controls
Contrast-specific imaging mode
B-mode
Contrast-specific imaging mode
B-mode
Contrast-specific imaging mode
B-mode

Set 3

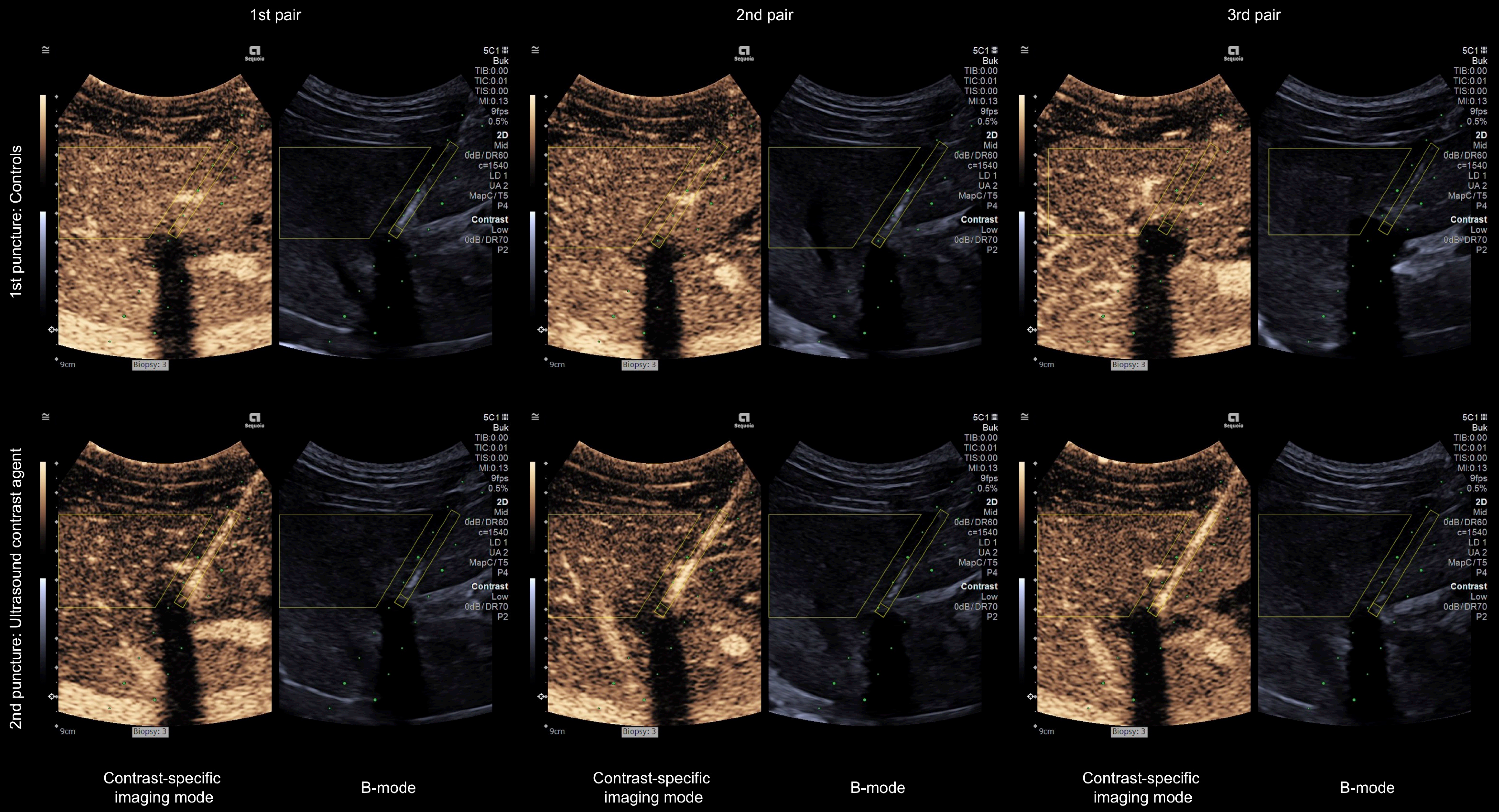
1st pair
2nd pair
3rd pair
1st puncture: Controls
2nd puncture: Ultrasound contrast agent
5C1
Buk
TIB:0.00
TIC:0.01
TIS:0.00
MI:0.13
9fps
0.5%
2D
Mid
0dB/DR60
c=1540
LD 1
UA 2
MapC/T5
P4
Contrast
Low
0dB/DR70
P2
9cm
Biopsy: 3
Contrast-specific imaging mode
B-mode
Contrast-specific imaging mode
B-mode
Contrast-specific imaging mode
B-mode

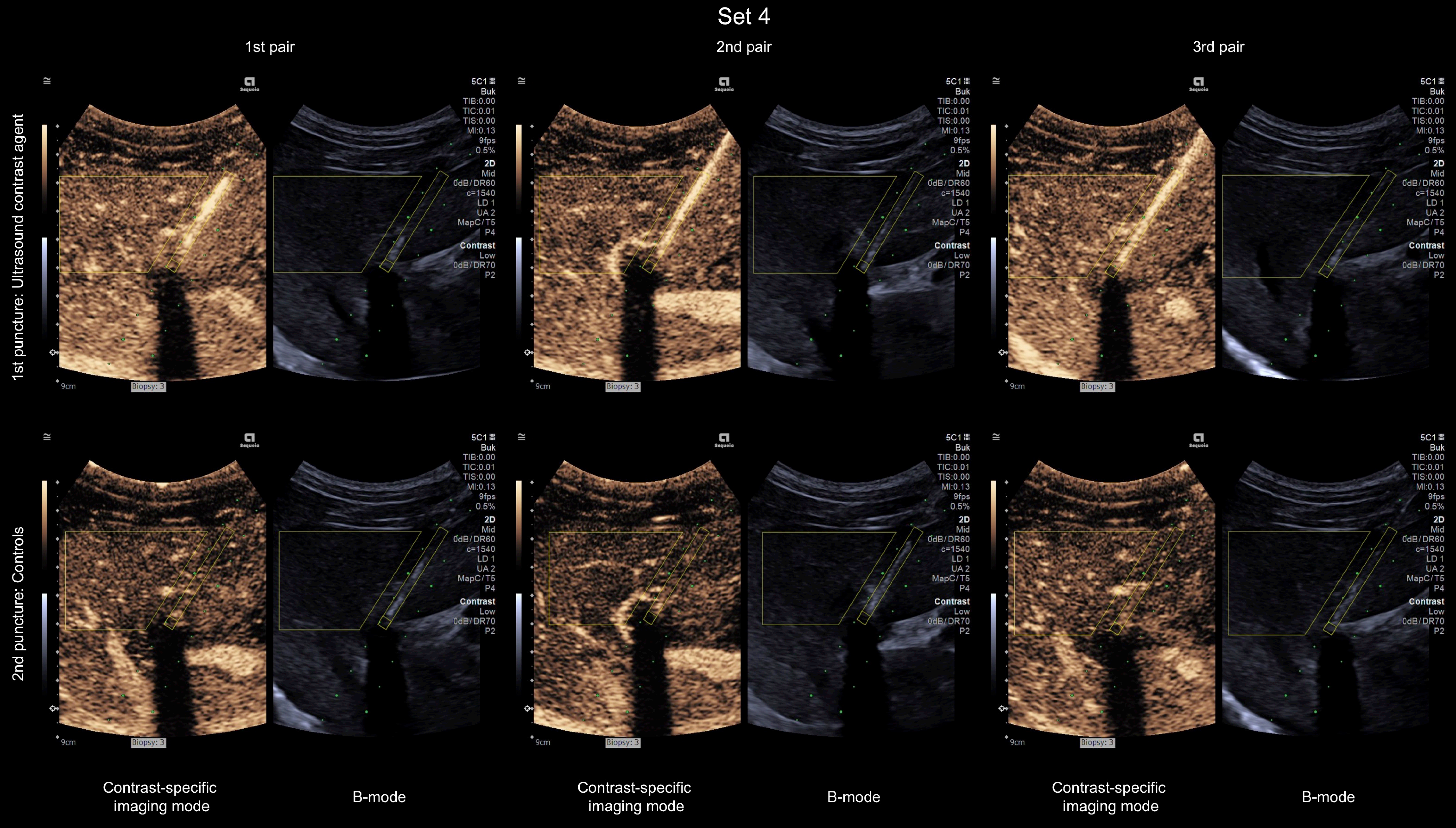
Set 4
1st pair
2nd pair
3rd pair
1st puncture: Ultrasound contrast agent
2nd puncture: Controls
5C1
Buk
TIB:0.00
TIC:0.01
TIS:0.00
MI:0.13
9fps
0.5%
2D
Mid
0dB/DR60
c=1540
LD 1
UA 2
MapC/T5
P4
Contrast
Low
0dB/DR70
P2
9cm
Biopsy: 3
Contrast-specific imaging mode
B-mode

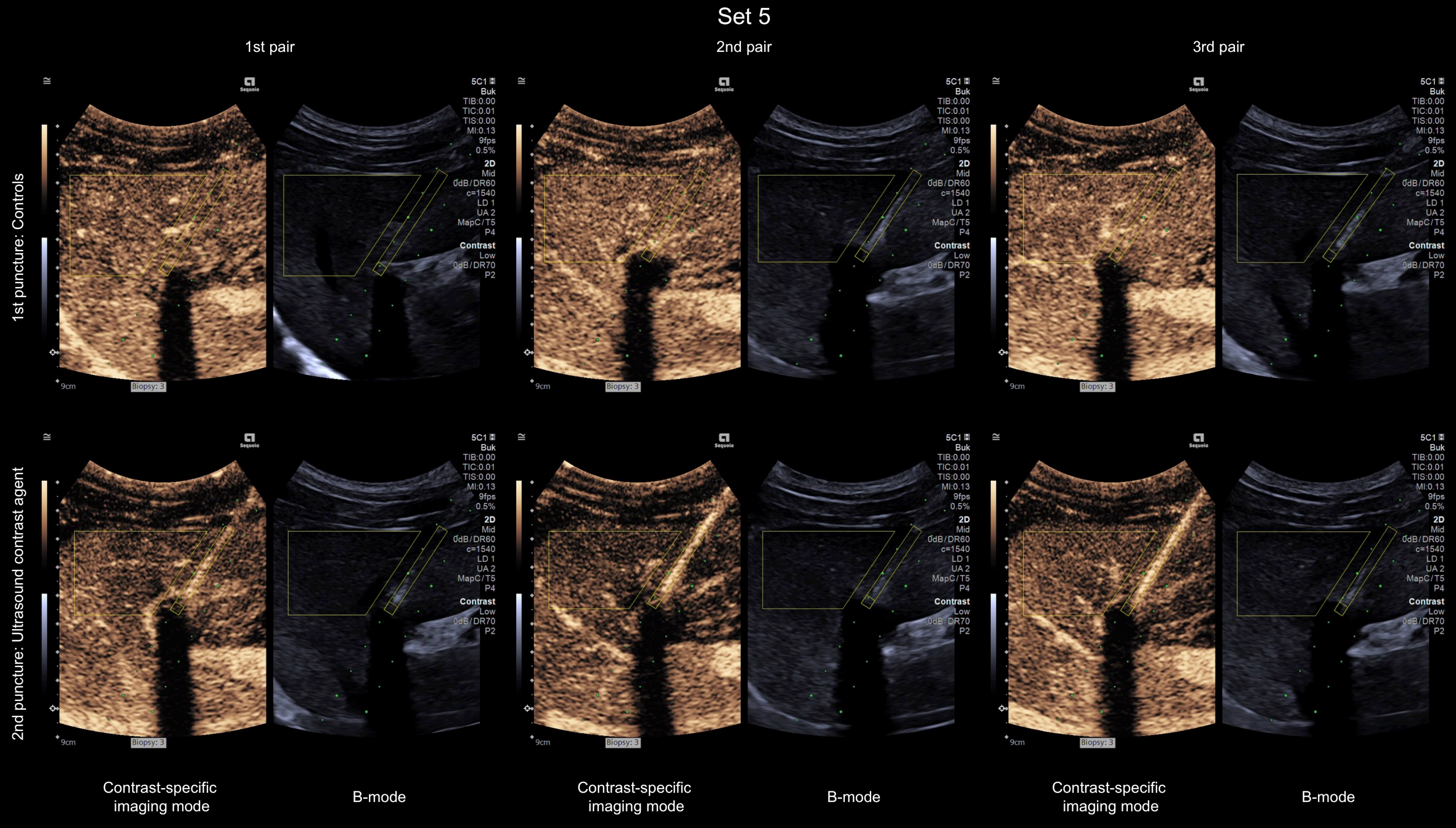
Set 5
1st pair
2nd pair
3rd pair
1st puncture: Controls
2nd puncture: Ultrasound contrast agent
Contrast-specific imaging mode
B-mode
Contrast-specific imaging mode
B-mode
Contrast-specific imaging mode
B-mode

Set 6

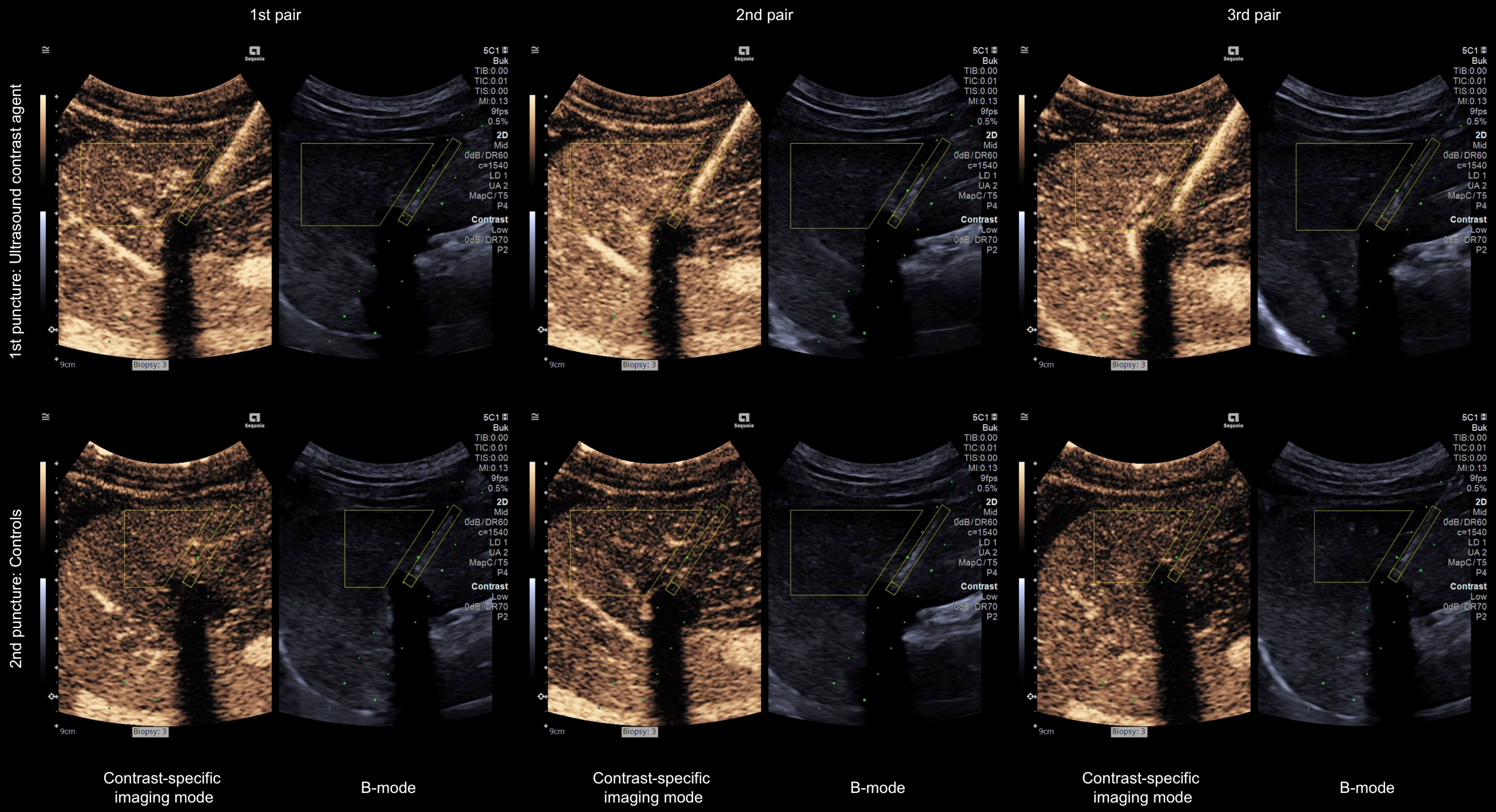
1st pair
2nd pair
3rd pair
1st puncture: Ultrasound contrast agent
2nd puncture: Controls
Contrast-specific imaging mode
B-mode
Contrast-specific imaging mode
B-mode
Contrast-specific imaging mode
B-mode

Set 7

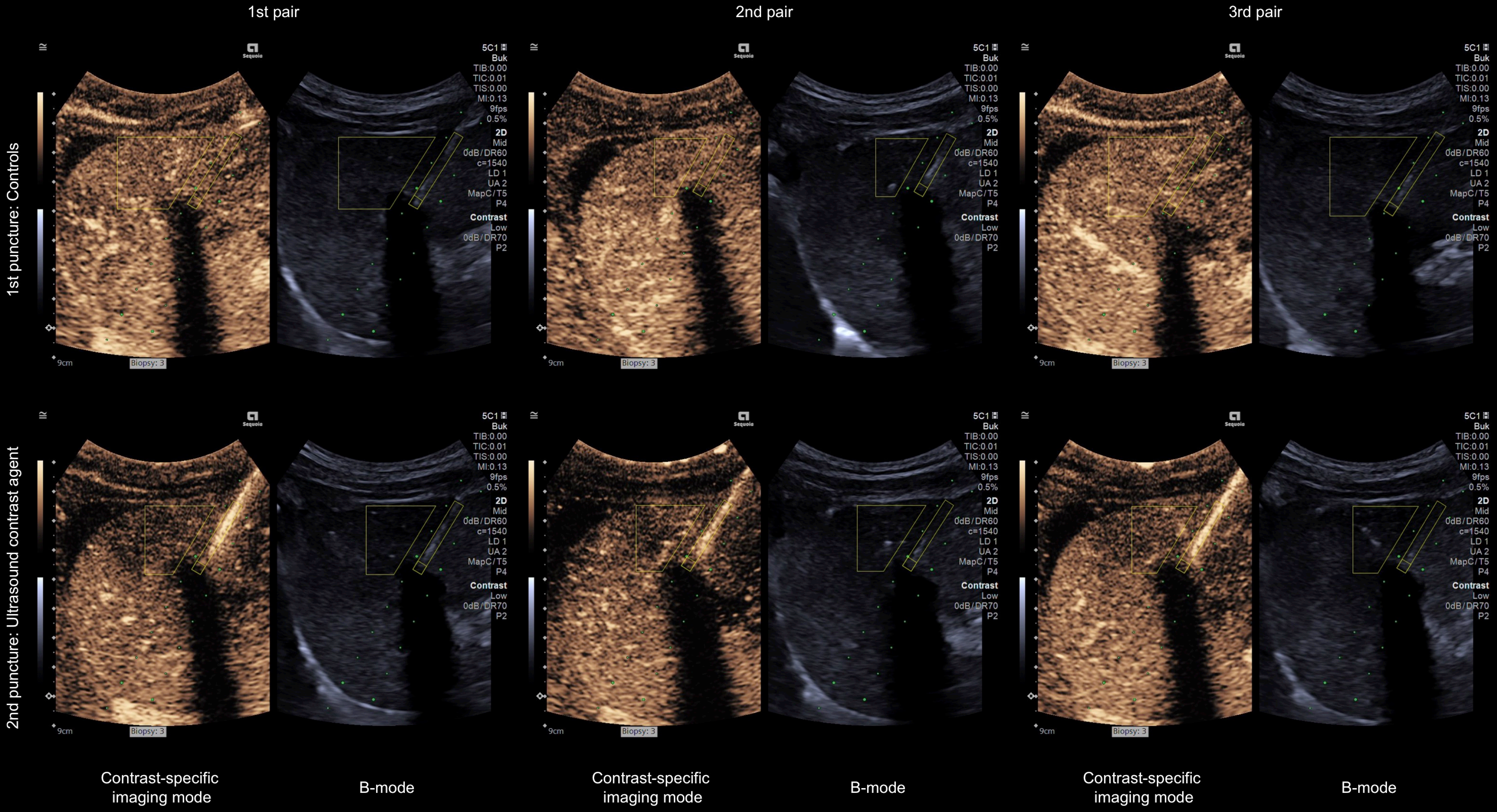
1st pair
2nd pair
3rd pair
1st puncture: Controls
2nd puncture: Ultrasound contrast agent
Contrast-specific imaging mode
B-mode
Contrast-specific imaging mode
B-mode
Contrast-specific imaging mode
B-mode
Biopsy: 3
9cm
5C1
Buk
TIB:0.00
TIC:0.01
TIS:0.00
MI:0.13
9fps
0.5%
2D
Mid
0dB/DR60
c=1540
LD 1
UA 2
MapC/T5
P4
Contrast
Low
0dB/DR70
P2

Set 8

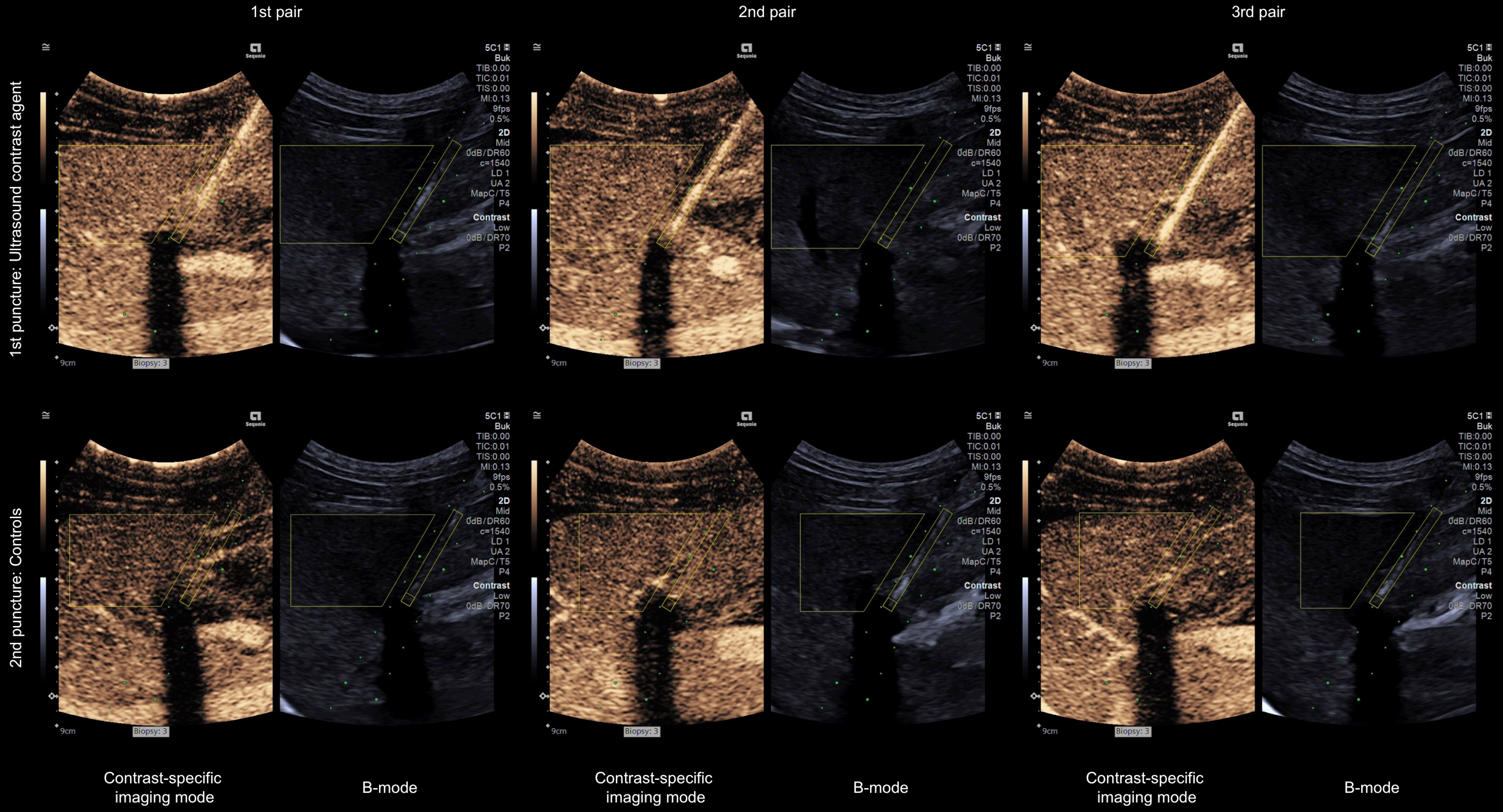

1st pair
2nd pair
3rd pair
1st puncture: Ultrasound contrast agent
2nd puncture: Controls
5C1
Buk
TIB:0.00
TIC:0.01
TIS:0.00
MI:0.13
9fps
0.5%
2D
Mid
0dB/DR60
c=1540
LD 1
UA 2
MapC/T5
P4
Contrast
Low
0dB/DR70
P2
9cm
Biopsy: 3
Contrast-specific imaging mode
B-mode
Contrast-specific imaging mode
B-mode
Contrast-specific imaging mode
B-mode

Set 9

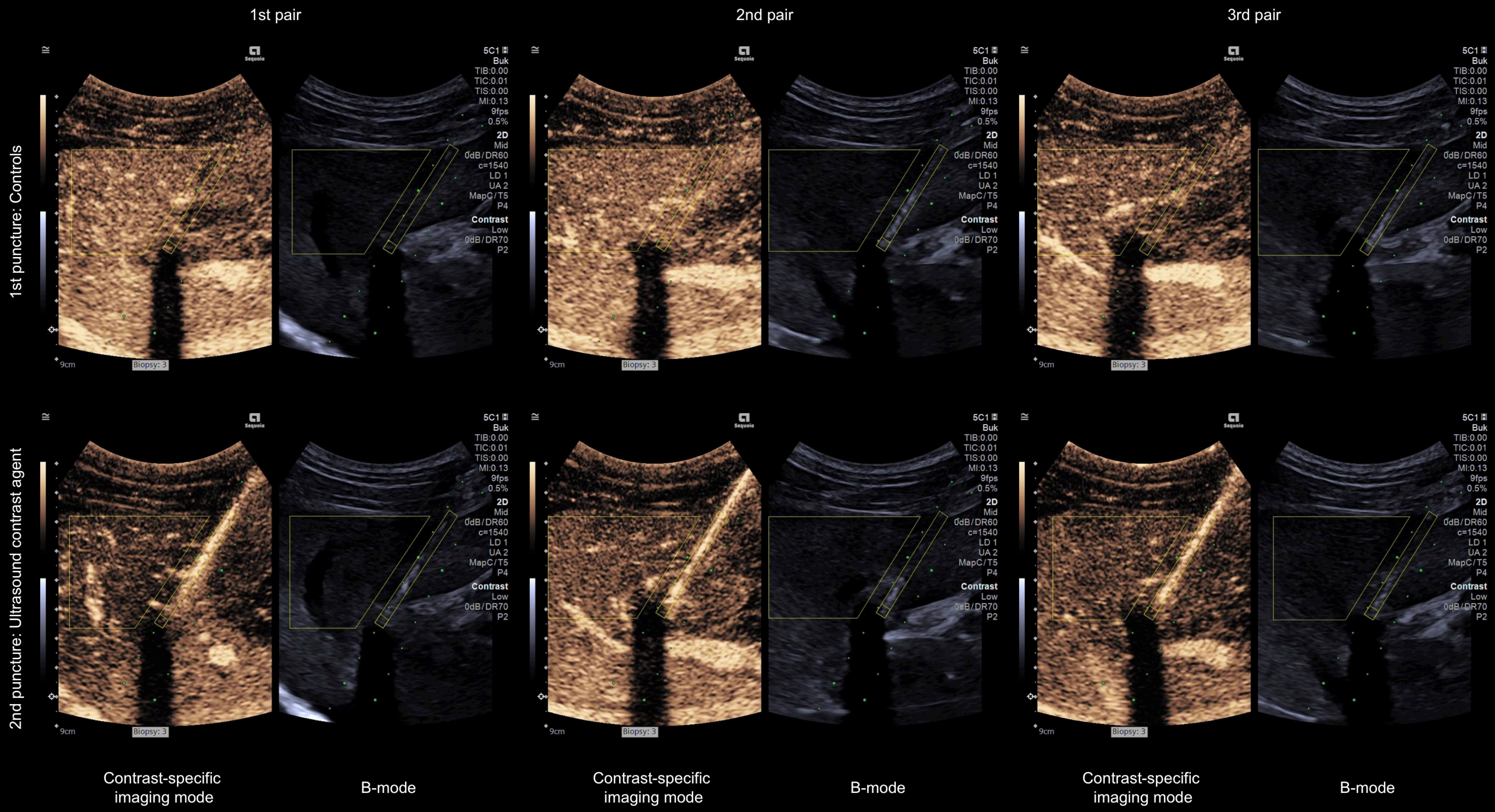
1st pair
2nd pair
3rd pair
1st puncture: Controls
2nd puncture: Ultrasound contrast agent
Contrast-specific imaging mode
B-mode
Contrast-specific imaging mode
B-mode
Contrast-specific imaging mode
B-mode

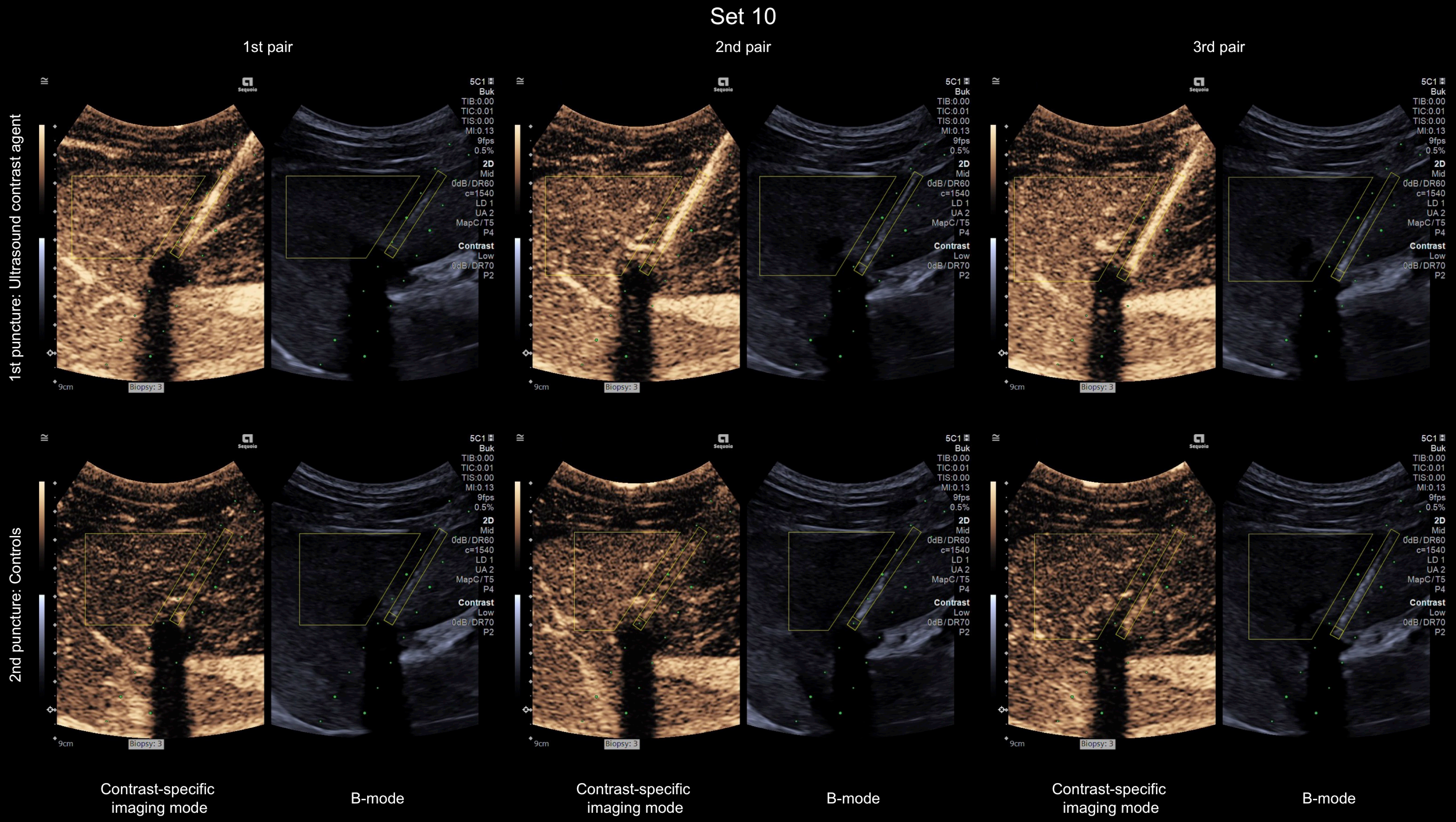
Set 10
1st pair
2nd pair
3rd pair
1st puncture: Ultrasound contrast agent
2nd puncture: Controls
Contrast-specific imaging mode
B-mode
Contrast-specific imaging mode
B-mode
Contrast-specific imaging mode
B-mode
