# Supplementary figures and images for: Ultrasound Contrast Agent Needle Priming: Impact on Sonographic Biopsy Needle Visibility in a Porcine Liver Model
Source: Cardiovasc Intervent Radiol. 2024 Jun 19;47(7):1000–8. doi: 10.1007/s00270-024-03758-1 (PMC11239778; doi:10.1007/s00270-024-03758-1)

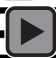

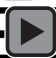

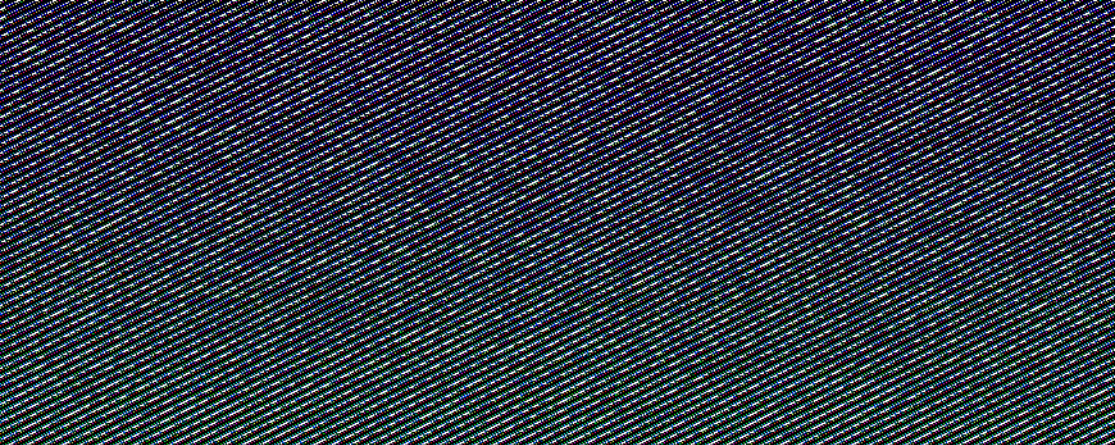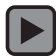

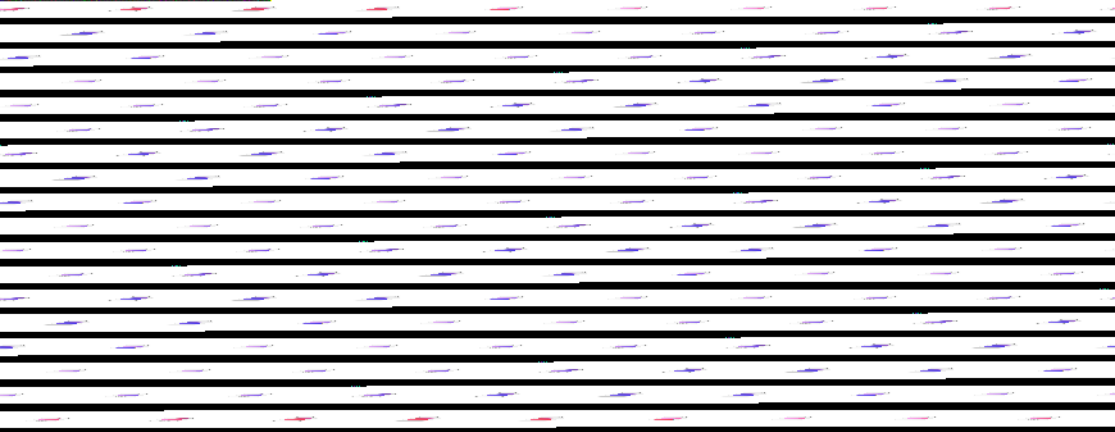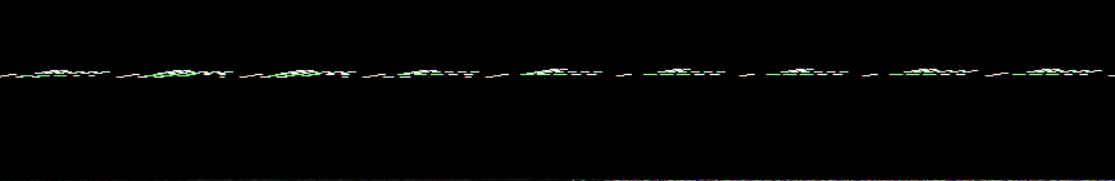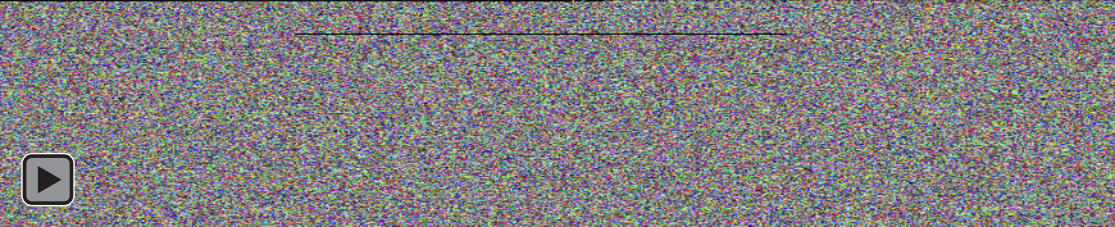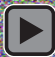

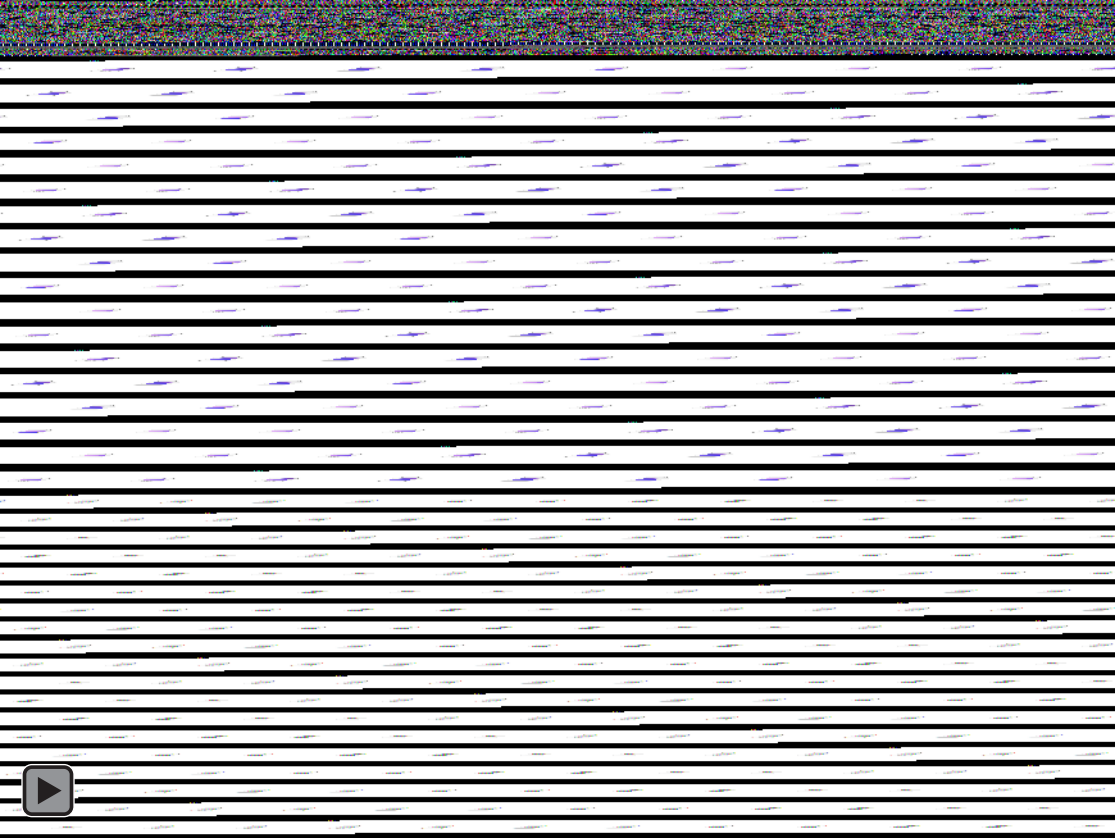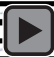

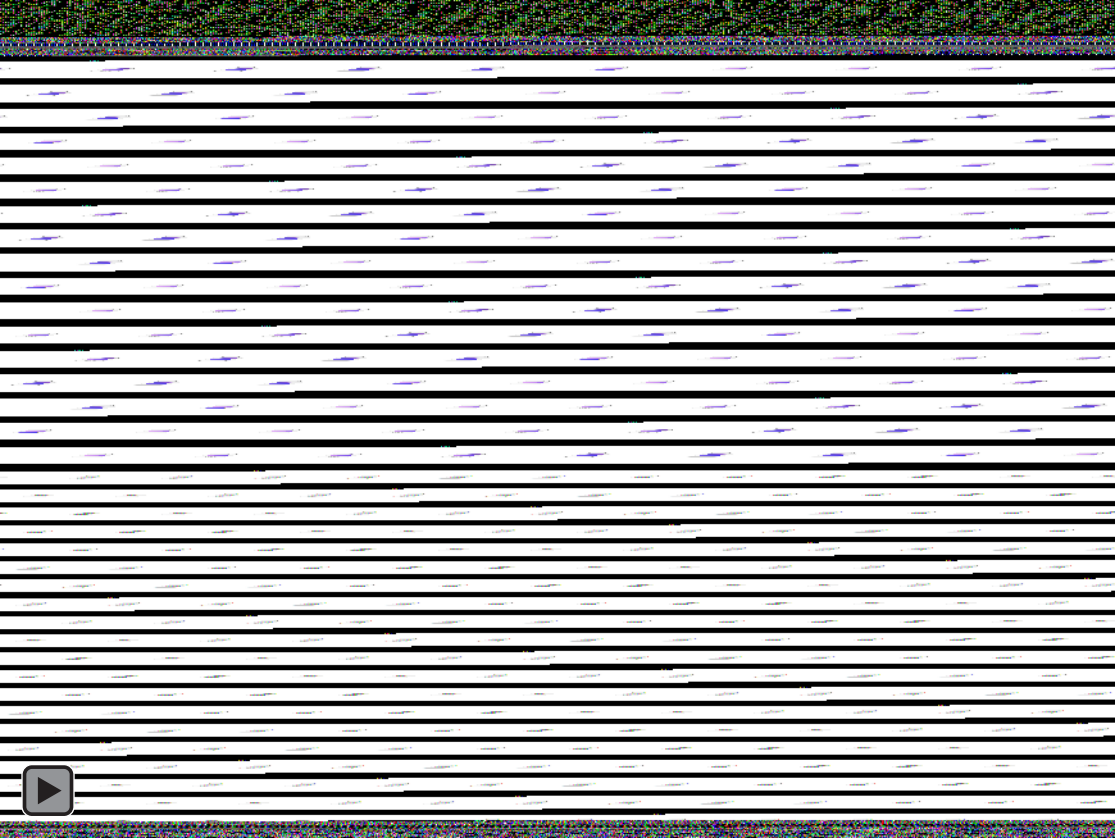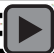

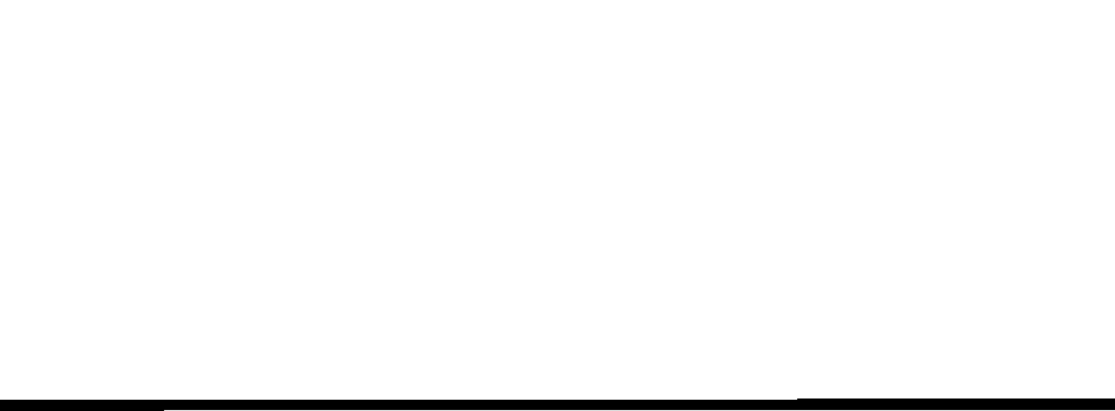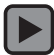

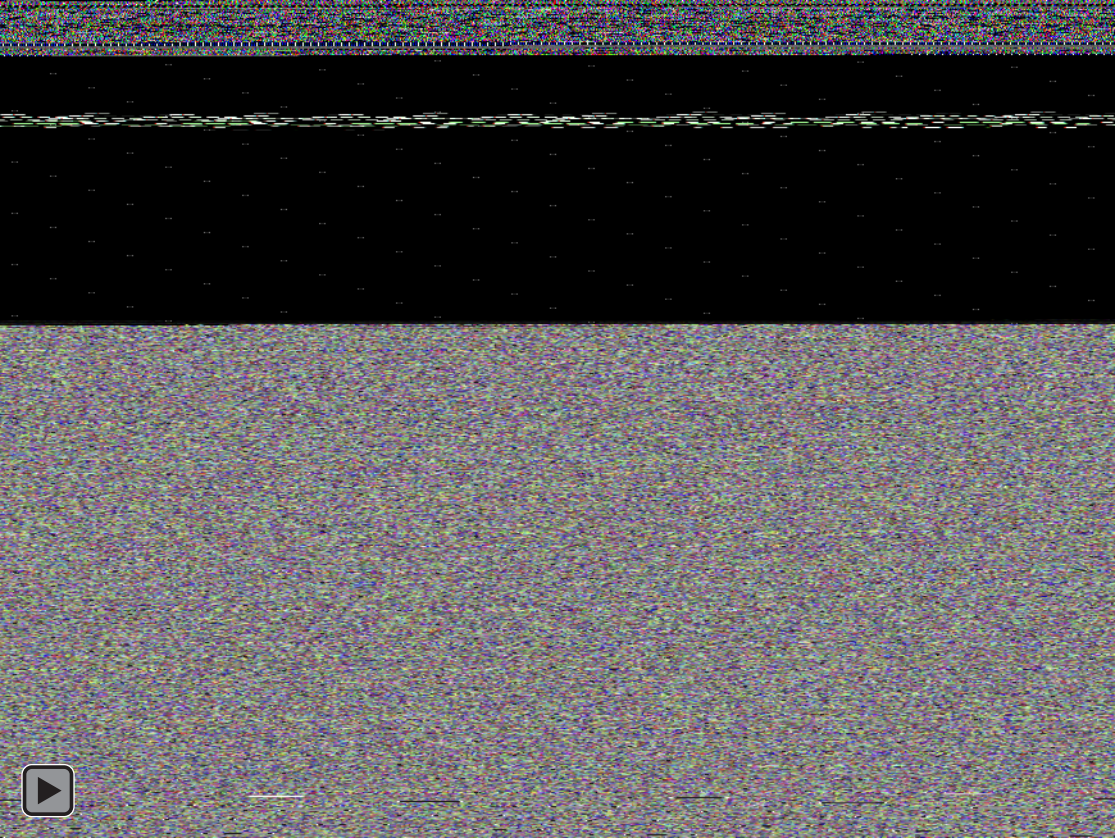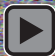

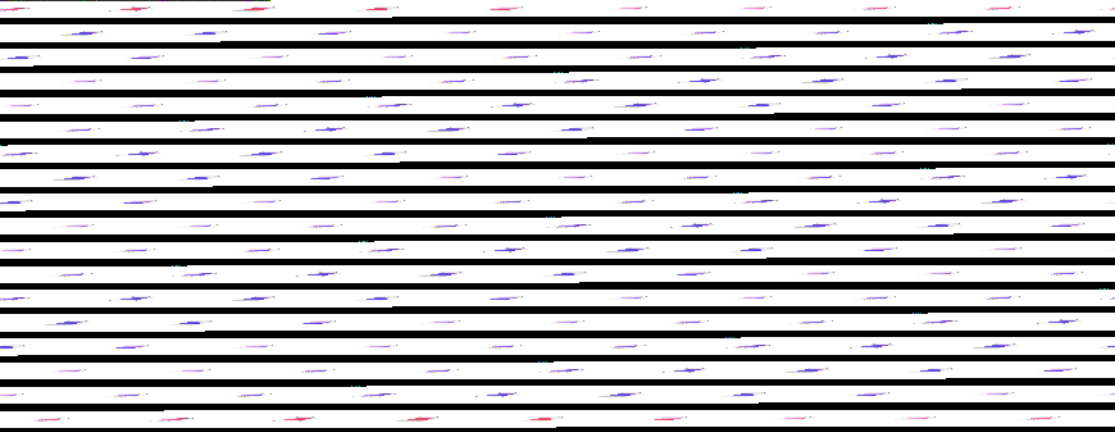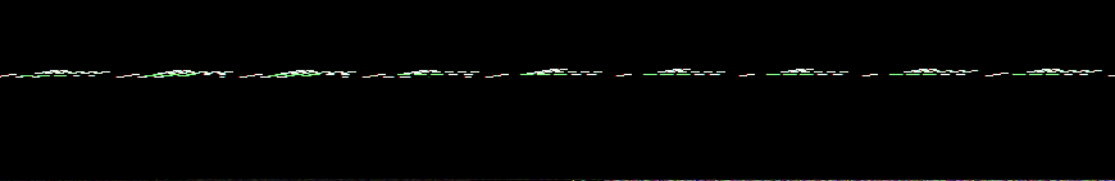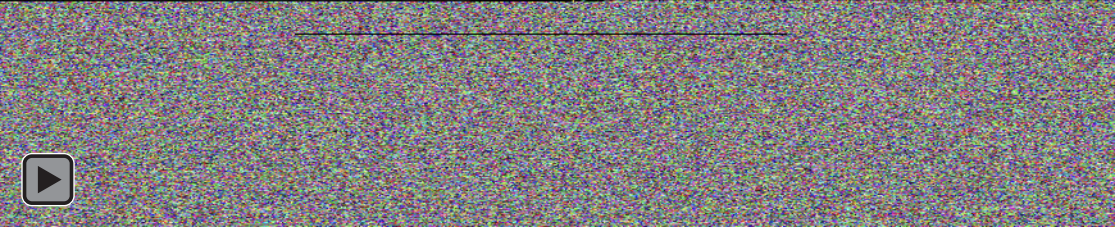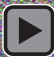

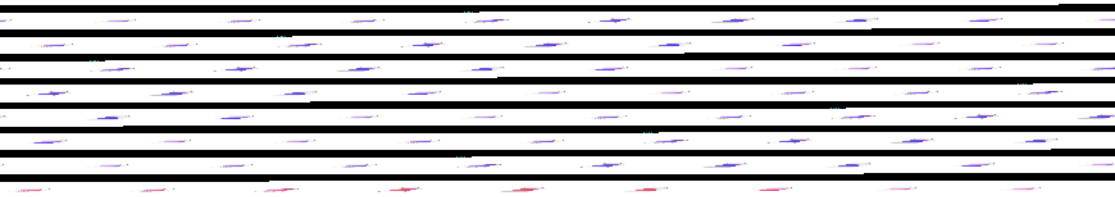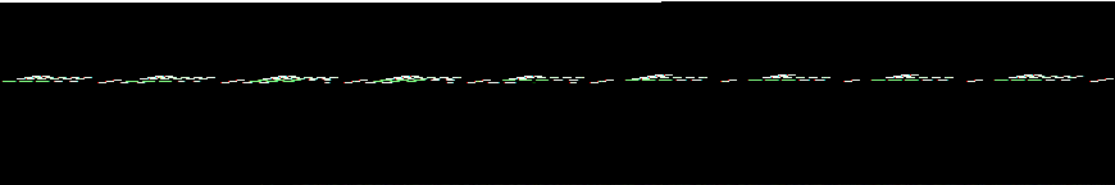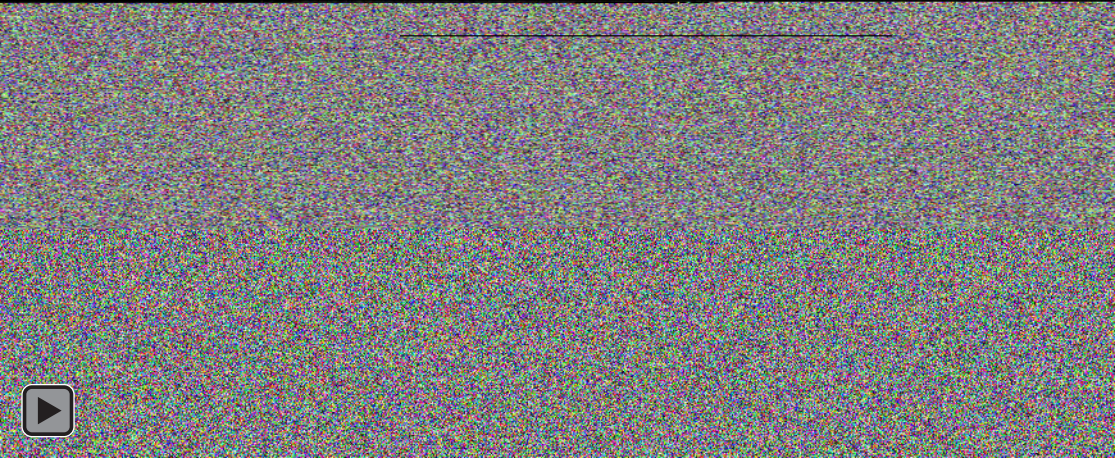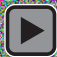

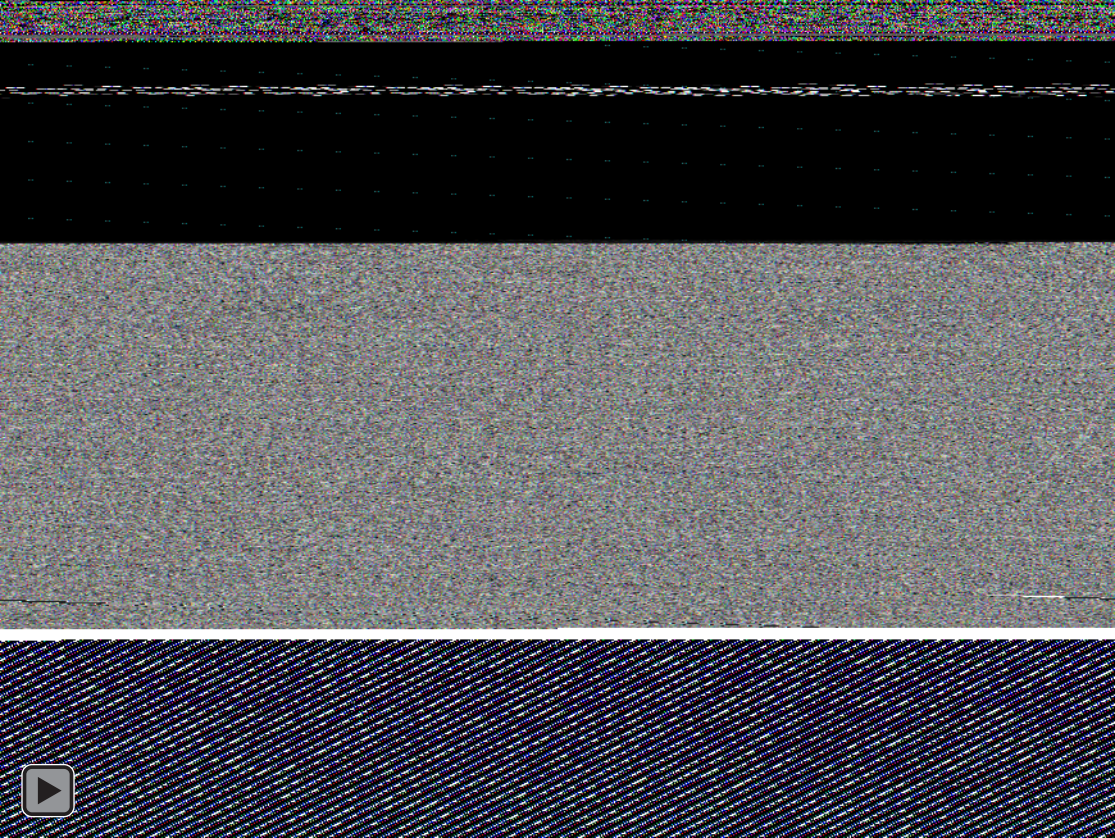

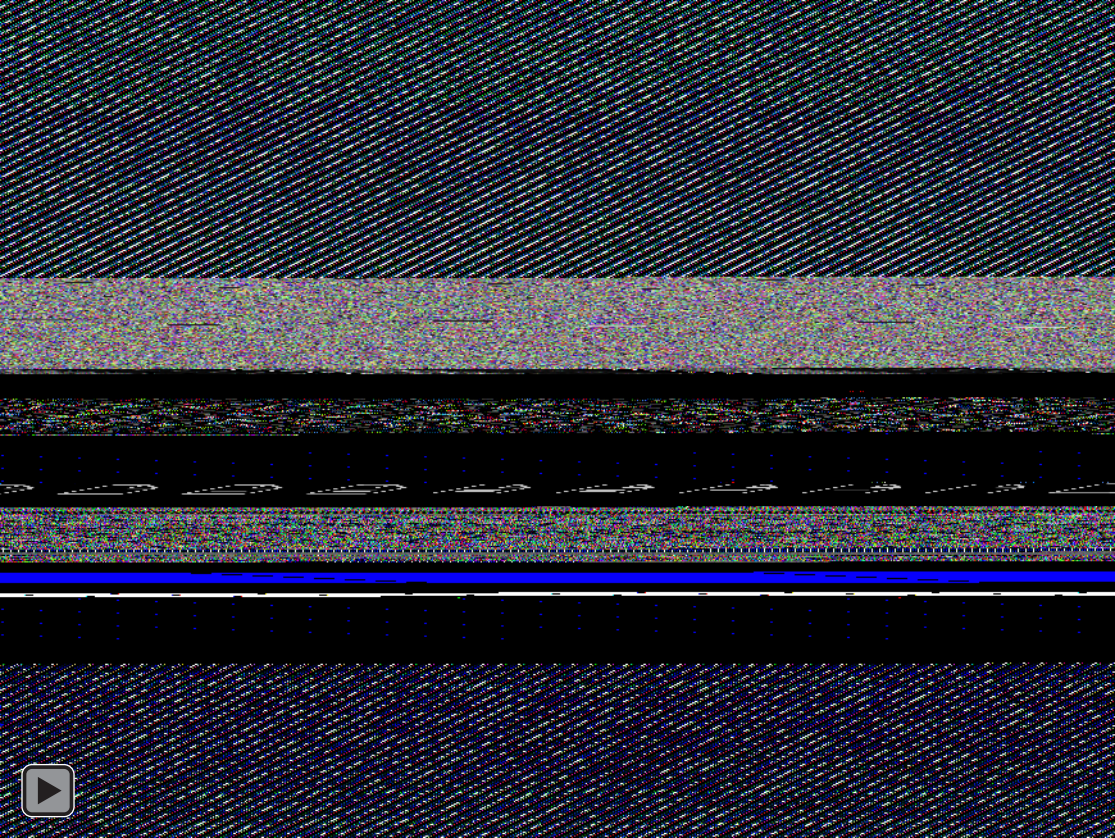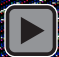

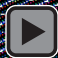

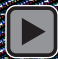

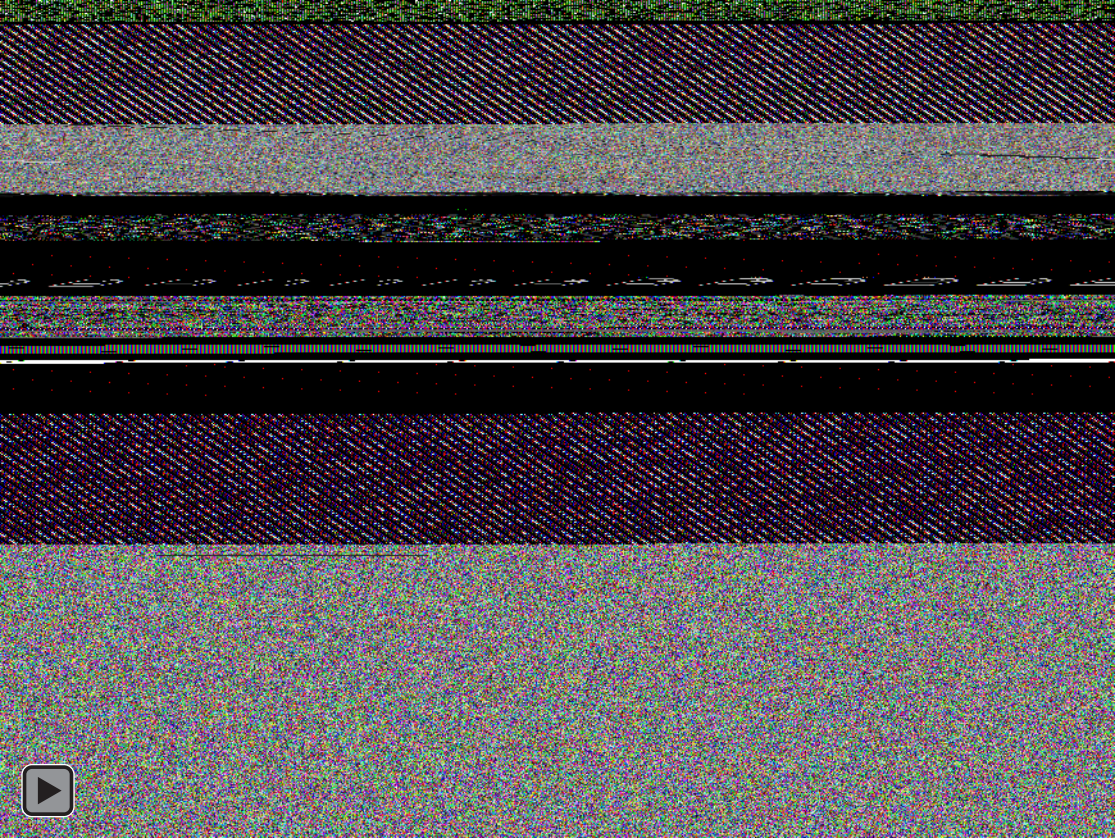

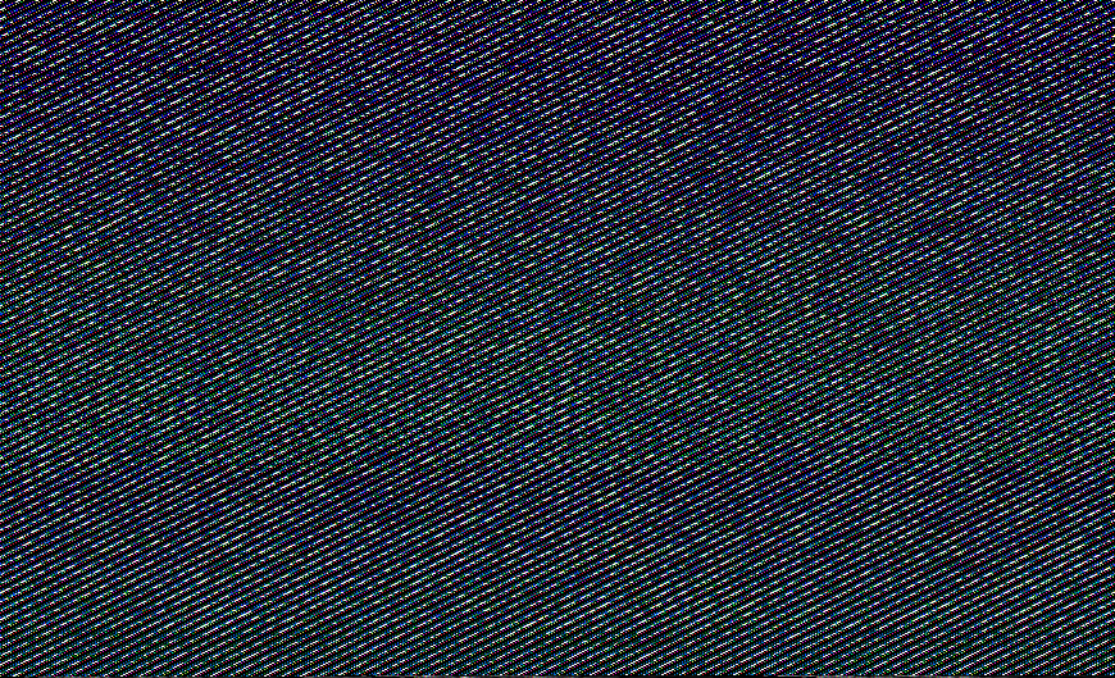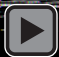

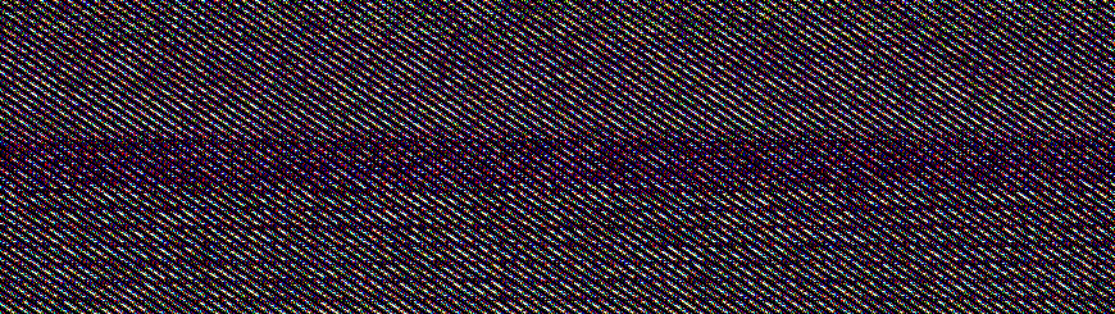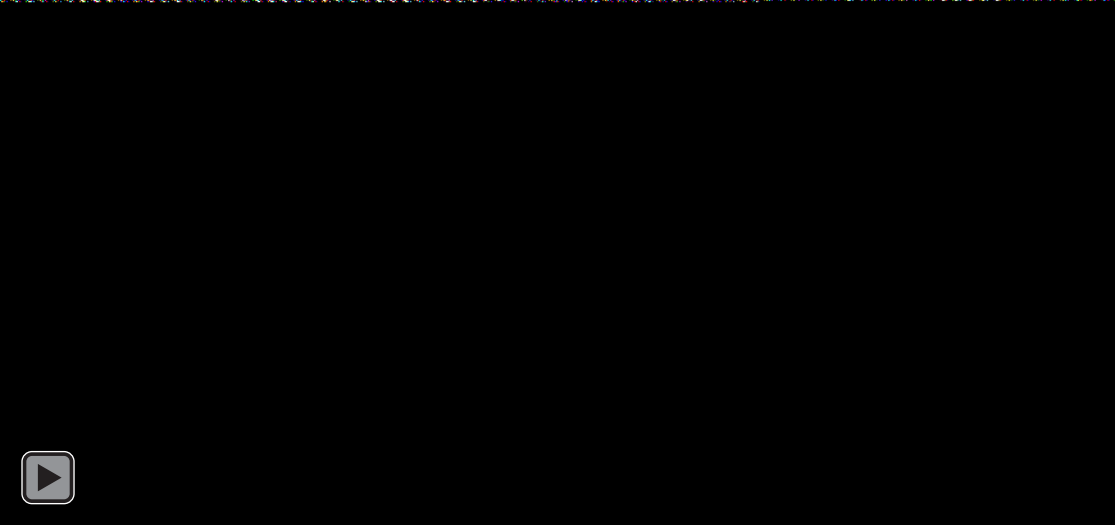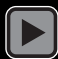

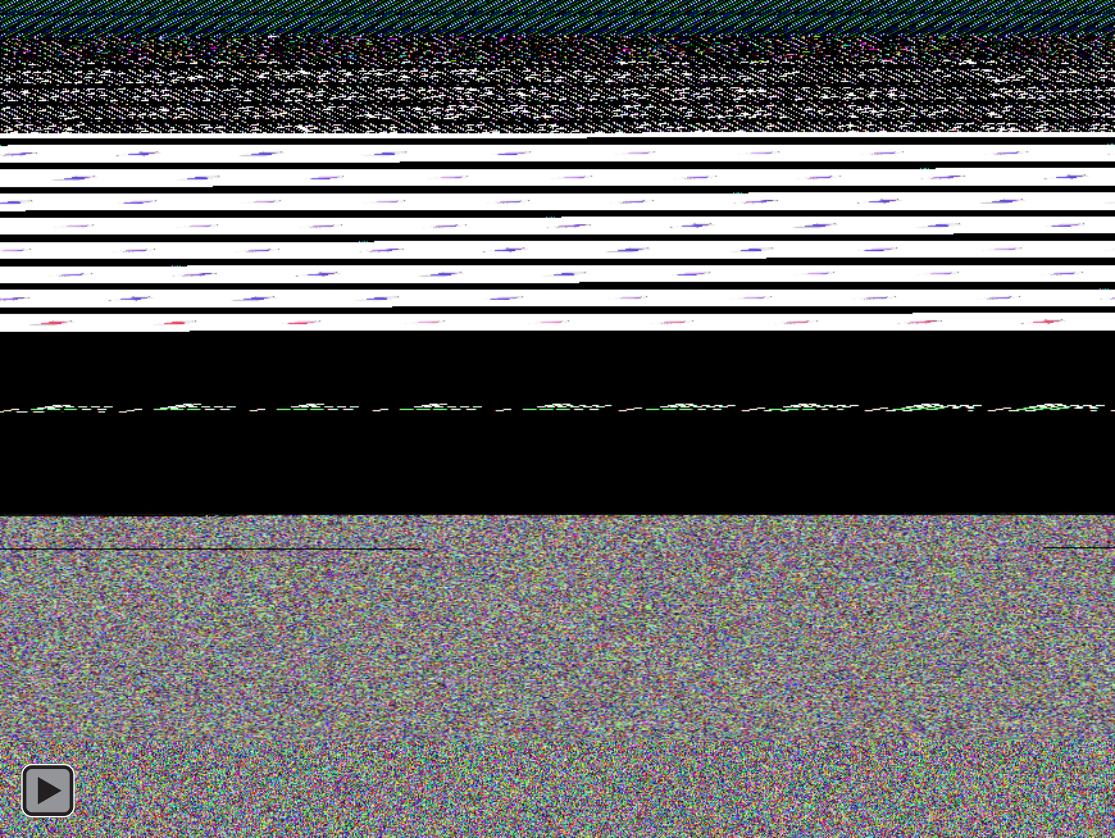

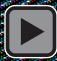

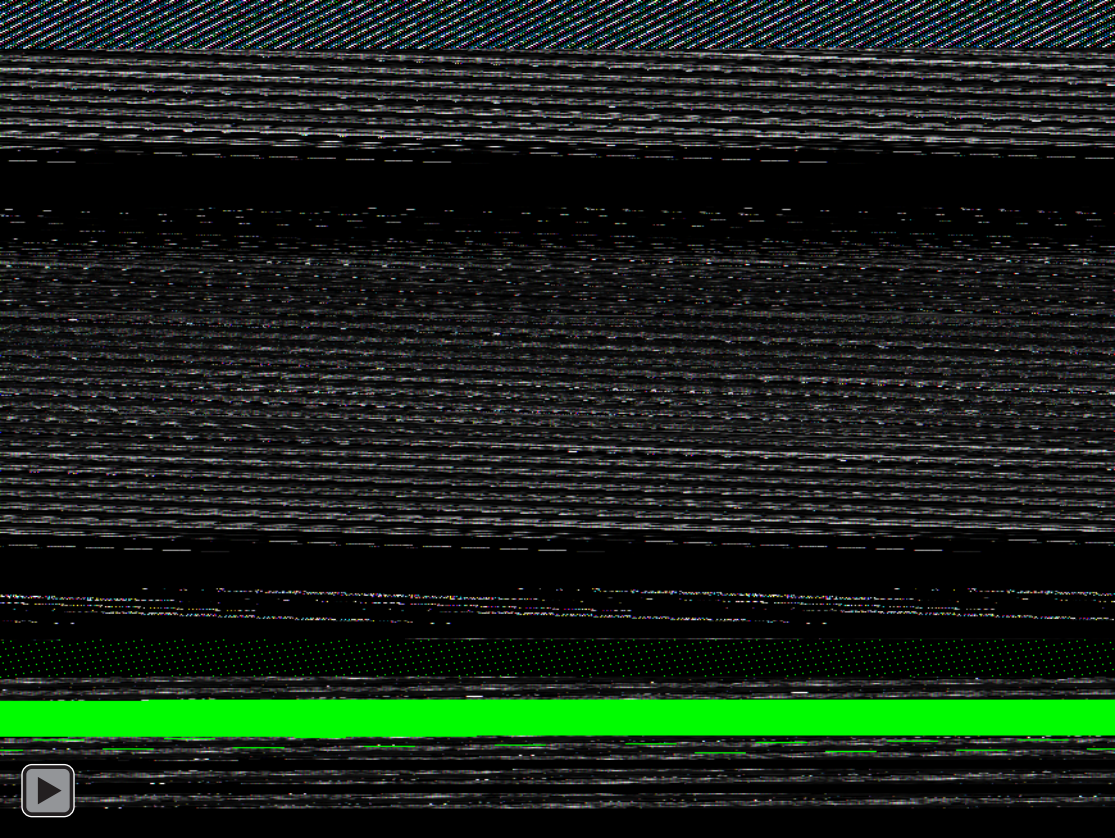

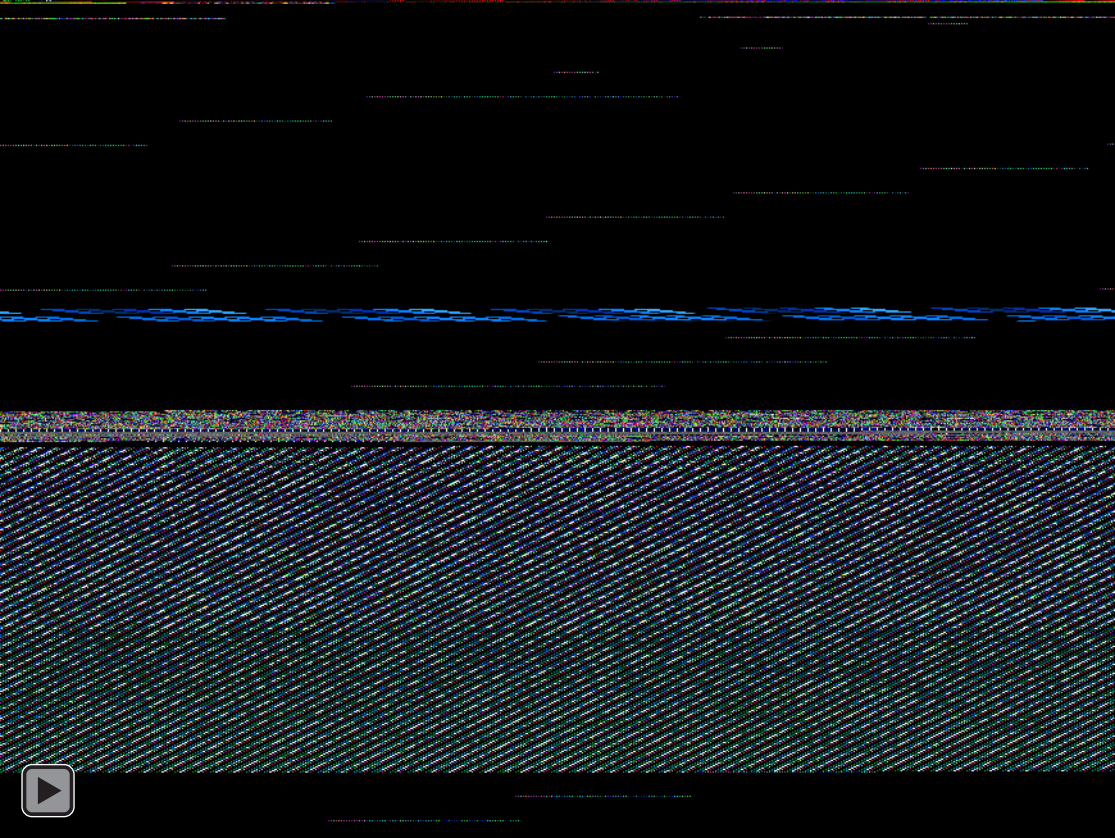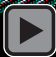

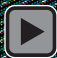

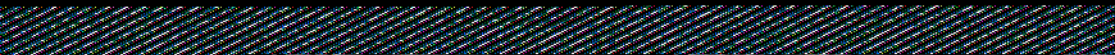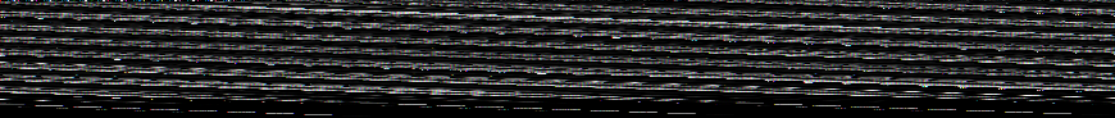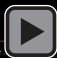

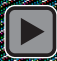

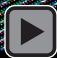

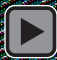

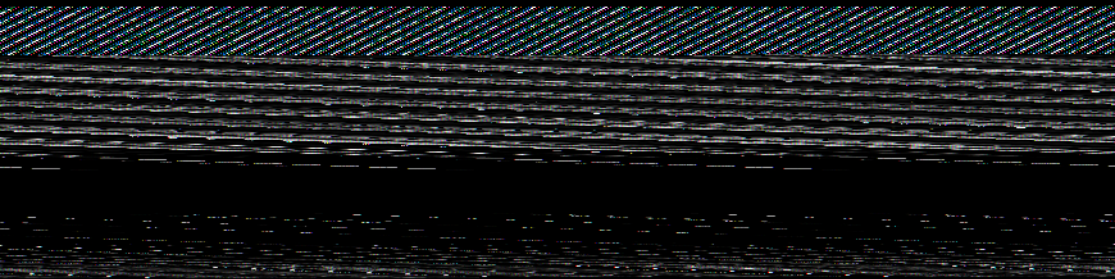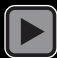

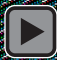

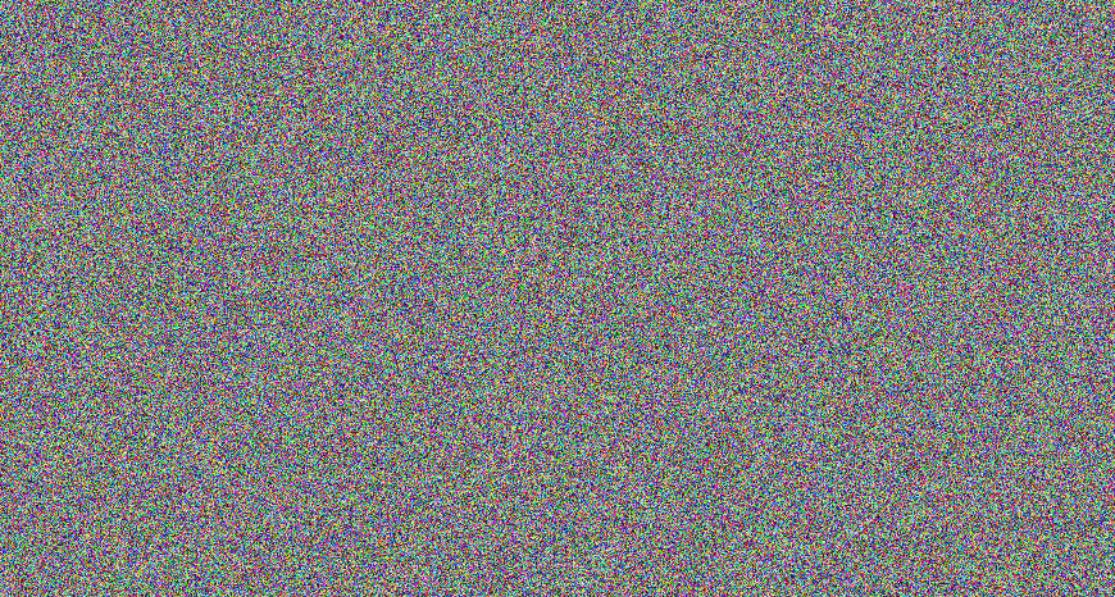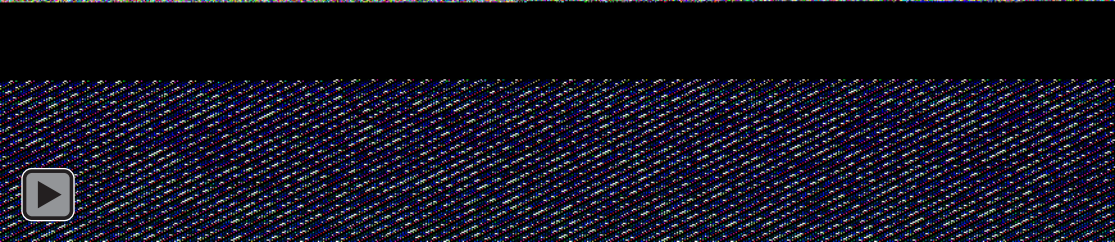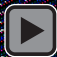

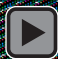

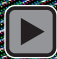

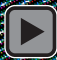

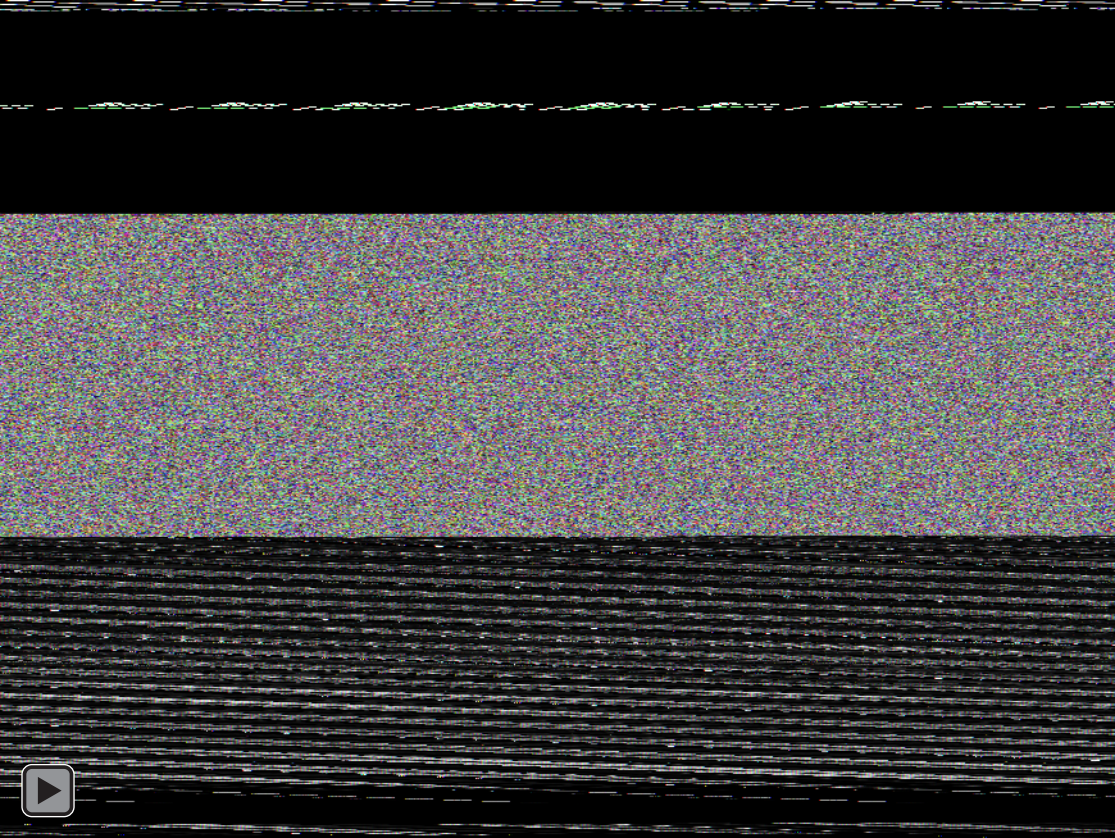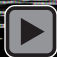

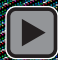

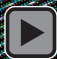

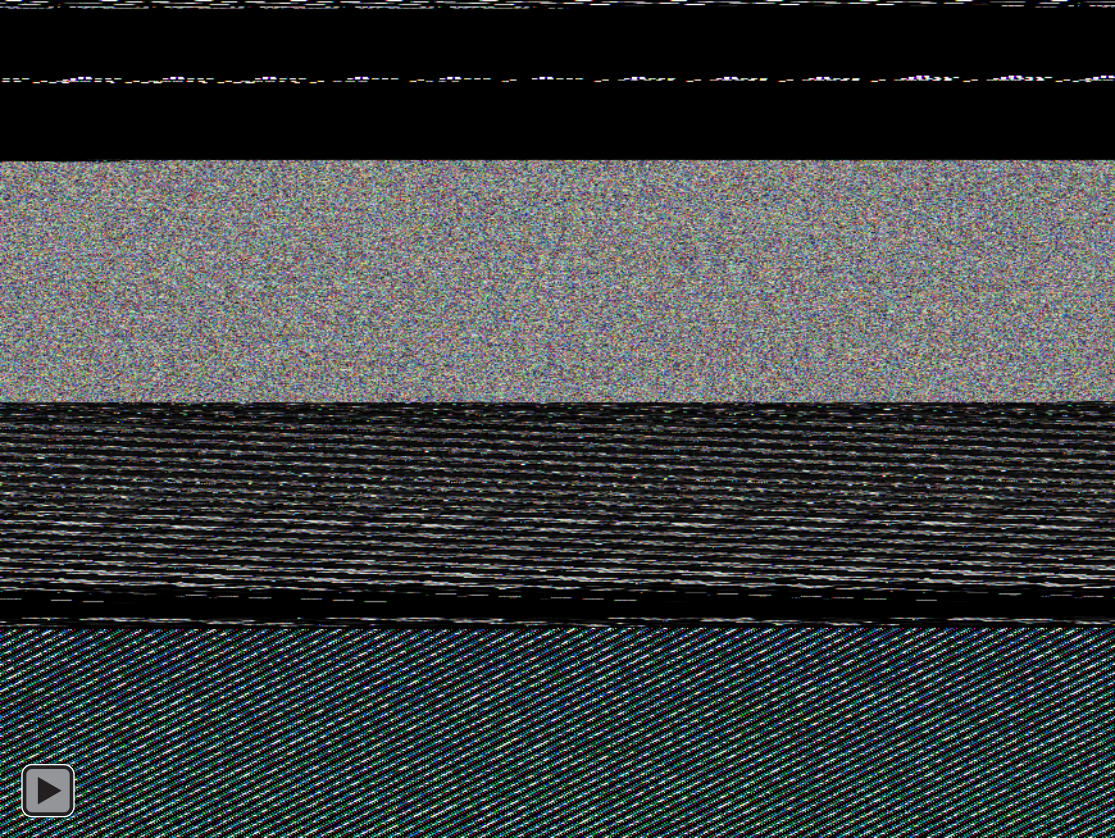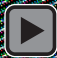

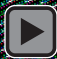

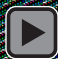

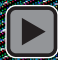

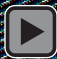

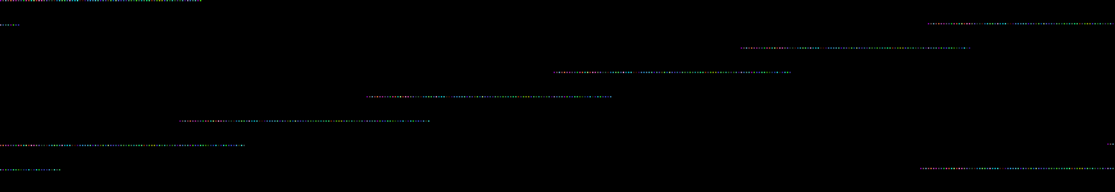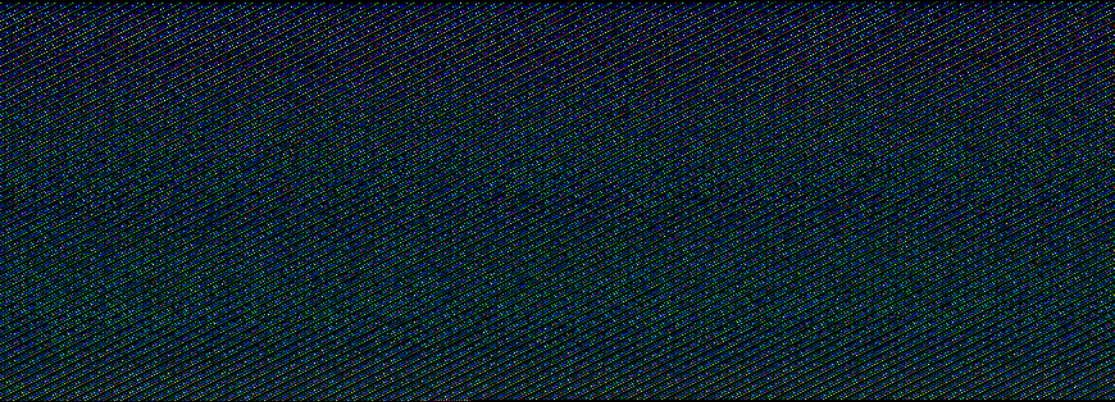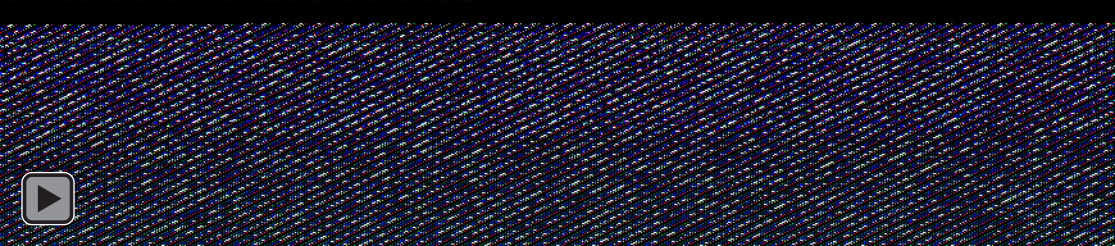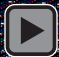

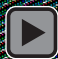

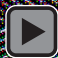

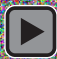

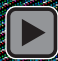

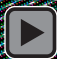

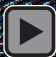

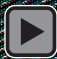

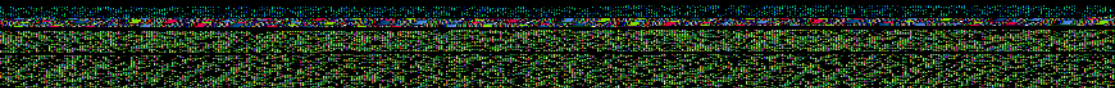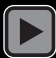

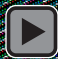

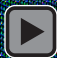

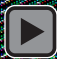

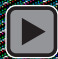

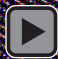

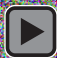

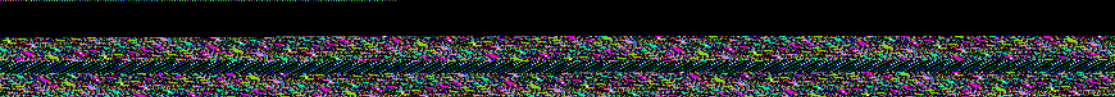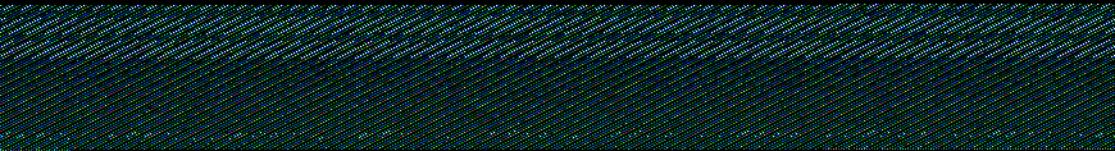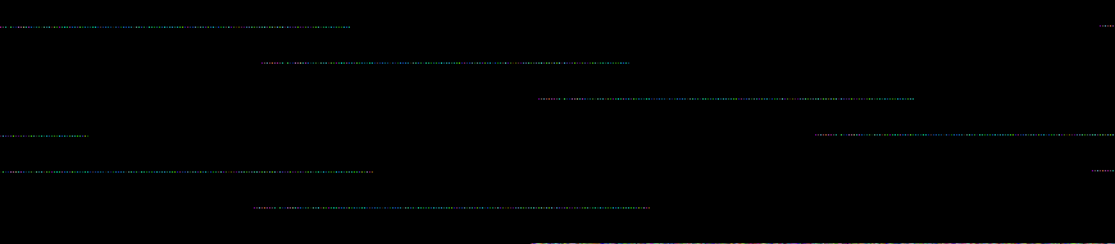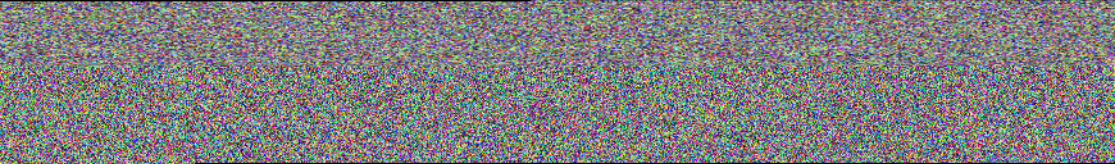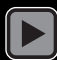

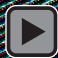

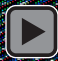

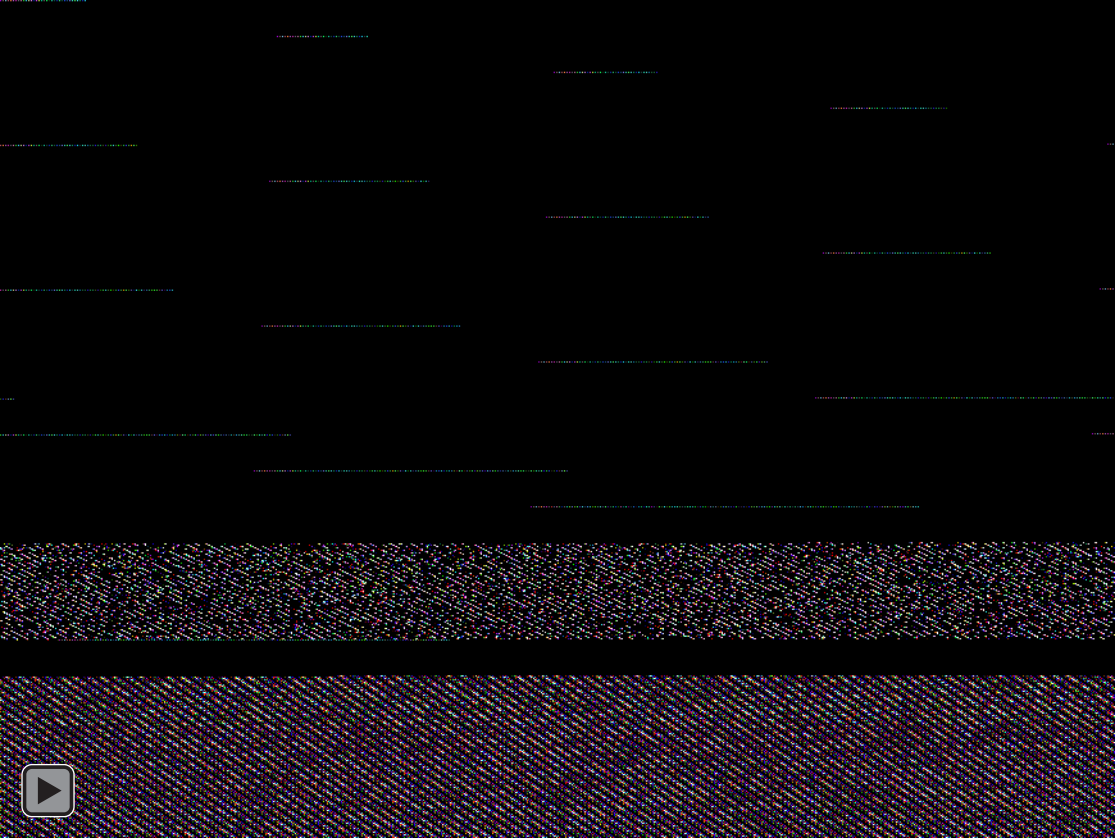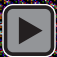

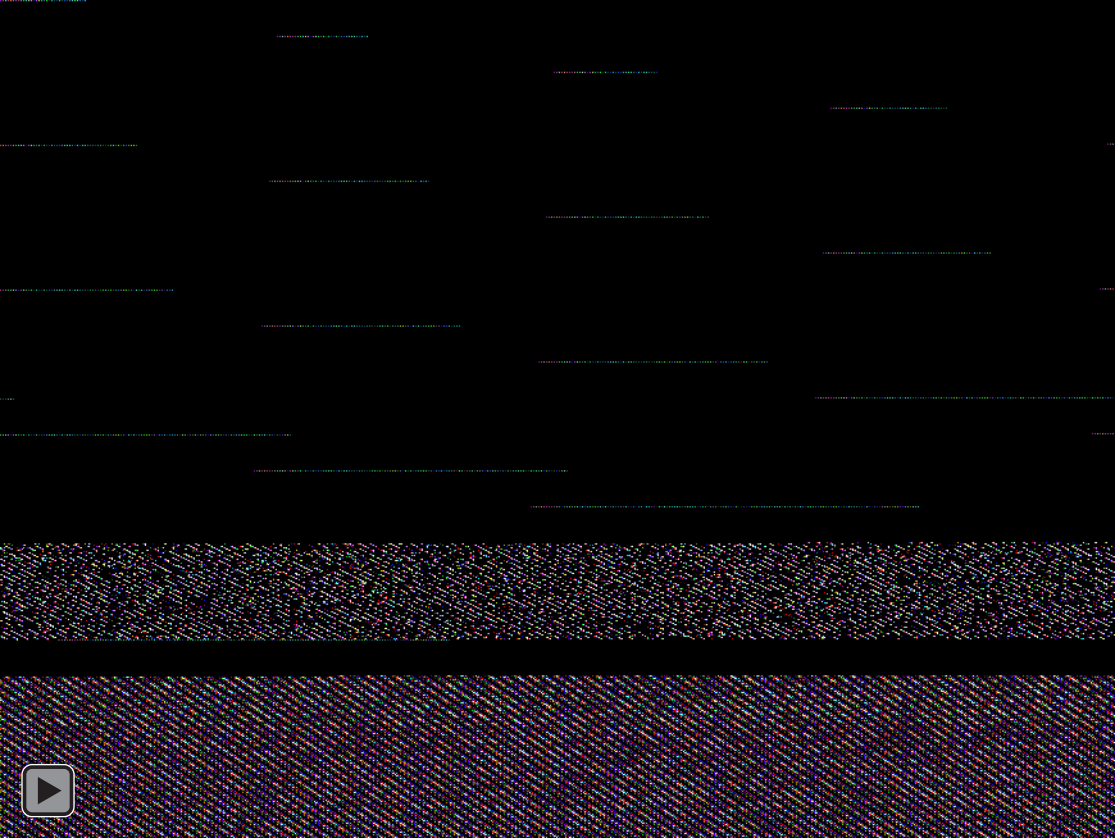

Supplement: Supplementary file 3 — Supplementary file3 (PDF 176759 KB) [file 270_2024_3758_MOESM3_ESM.pdf]
